# The Consumption of Cholesterol-Enriched Diets Conditions the Development of a Subtype of HCC with High Aggressiveness and Poor Prognosis

**DOI:** 10.3390/cancers13071721

**Published:** 2021-04-06

**Authors:** Arturo Simoni-Nieves, Soraya Salas-Silva, Lisette Chávez-Rodríguez, Alejandro Escobedo-Calvario, Matthis Desoteux, Leticia Bucio, Verónica Souza, Roxana U. Miranda-Labra, Linda E. Muñoz-Espinosa, Cédric Coulouarn, María Concepción Gutiérrez-Ruiz, Jens U. Marquardt, Luis E. Gomez-Quiroz

**Affiliations:** 1 Posgrado en Biología Experimental, DCBS, Universidad Autónoma Metropolitana Iztapalapa, Mexico City 09340, Mexico; bioexpp22@gmail.com (A.S.-N.); esss2491@gmail.com (S.S.-S.); lisettechavez23@gmail.com (L.C.-R.); escobedocoa@hotmail.com (A.E.-C.); 2 Área de Medicina Experimental y Traslacional, Departamento de Ciencias de la Salud, Universidad Autónoma Metropolitana-Iztapalapa, Mexico City 09340, Mexico; lebo@xanum.uam.mx (L.B.); veso@xanum.uam.mx (V.S.); roxml@xanum.uam.mx (R.U.M.-L.); mcgr@xanum.uam.mx (M.C.G.-R.); 3 Centre de Lutte contre le Cancer Eugène Marquis, Inserm, Univ Rennes, COSS (Chemistry, Oncogenesis Stress Signaling), UMR_S 1242, 35042 Rennes, France; desoteux.matthis@hotmail.fr (M.D.); cedric.coulouarn@inserm.fr (C.C.); 4 Laboratorio de Medicina Experimental, Unidad de Medicina Traslacional, IIB, UNAM/Instituto Nacional de Cardiología Ignacio Chavez, Mexico City 14080, Mexico; 5 Liver Unit, Department of Internal Medicine, “Dr. José E. González” University Hospital, Universidad Autónoma de Nuevo León, Monterrey 64460, NL, Mexico; linda_uanl@hotmail.com; 6 Department of Medicine I, University Hospital Schleswig-Holstein, 23562 Lübeck, Germany; Jens.Marquardt@uksh.de

**Keywords:** NAFLD, NASH, HCC, cholesterol, Western diet

## Abstract

**Simple Summary:**

It is well known that non-alcoholic fatty liver disease is an important risk factor in the development of hepatocellular carcinoma, but the implication of cholesterol in this subject remains unclear, especially in western countries where its consumption is particularly elevated. This work provides evidence of a cholesterol-related transcriptional fingerprint and its implications in the progression and aggressiveness of hepatocellular carcinoma with remarkable interest in clinical practice.

**Abstract:**

Non-alcoholic fatty liver disease (NAFLD) and progression to non-alcoholic steatohepatitis (NASH) result as a consequence of diverse conditions, mainly unbalanced diets. Particularly, high-fat and cholesterol content, as well as carbohydrates, such as those commonly ingested in Western countries, frequently drive adverse metabolic alterations in the liver and promote NAFLD development. Lipid liver overload is also one of the main risk factors for initiation and progression of hepatocellular carcinoma (HCC), but detailed knowledge on the relevance of high nutritional cholesterol remains elusive. We were aimed to characterize HCC development in mice fed with a Western diet (high in lipids and cholesterol) and to identify molecular alterations that define a subtype of liver cancer induced by lipid overload. Mice under western or high cholesterol diets more frequently developed tumors with a more aggressive phenotype than animals fed with a chow diet. Associated changes involved macrophage infiltration, angiogenesis, and stemness features. RNA-seq revealed a specific gene expression signature (*Slc41a; Fabp5; Igdcc4* and *Mthfd1l*) resembling the adverse phenotypic features and poor clinical outcomes seen in patients with HCC. In conclusion; consumption of lipid enriched diets; particularly cholesterol; could accelerate HCC development with an aggressive phenotype and poor prognosis

## 1. Introduction

Non-alcoholic fatty liver disease (NAFLD) is the fastest-growing chronic liver disease [1]. NAFLD and its inflammatory progressive form non-alcoholic steatohepatitis (NASH) are part of the natural history of hepatic diseases. It is well-known that changes in dietary behaviors contribute to the initiation and progression of this lipid-overload condition, increasing the incidence of liver cancer. The global prevalence of NAFLD is currently estimated to be 24% [2]. Accumulated evidence has suggested a significant association between NAFLD and liver cancer [3,4]. Hepatocellular carcinoma (HCC) is the main primary liver cancer; it represents the fifth and ninth cause of cancer-related death in males and females, respectively [5]. A global increase in the incidence of HCC is expected in the next 10–20 years [6]. HCC exhibits highly variable clinical outcomes, the prognostic variability of individuals with HCC supports the notion that HCC comprises several biologically distinct subtypes [7]. Compared with viral or alcoholic hepatitis, NAFLD is becoming a major etiology for HCC, based on the increasing prevalence of obesity, especially in Western countries [8]. A large-scale epidemiological study revealed that obesity could represent the main risk factor in cancer, such as HCC [9]. An effective molecular understanding of NASH-related HCC is urgently required for better management of patients with HCC, including early diagnosis and a more accurate prognosis, along with the design of optimized treatments. Most patients with NAFLD remain asymptomatic, and about 20–25% progress to developing a more severe chronic hepatic inflammatory disease, defined as NASH. Eventually 2.6% of those patients develop HCC [5,10]. HCC not only develops on a background of liver cirrhosis, but also on non-cirrhotic liver in NASH. The molecular events involved in NASH progression and HCC development are not fully understood [11]. Among the biochemical determinants characterizing the NAFLD are the accumulation of triglycerides and cholesterol. Particularly, cholesterol overload represents a key insult in the liver, as we and others have reported [12], and it has been associated with HCC development [3,4,13,14]. Although the relationship between NAFLD and HCC has been extensively reported [15], the particular contribution of cholesterol in HCC onset and progression has been poorly studied, especially in populations where dietary cholesterol intake is important. The cholesterol content in the Western diet is 10-fold higher than in the Mediterranean geographical zone [2]. Cholesterol is fundamental for life; it is the precursor of many hormones and bile salts, which are necessary for proper digestion. It is a remarkable component of lipid rafts, so changes in cholesterol levels seriously affect cell signaling, especially for growth factors or cytokine receptors [16]. A high cholesterol diet causes an accumulation of lipids in the hepatocytes, which are key components of lipoproteins used for lipid exportation [17]. Multiple mechanisms contribute to creating a favorable environment for the development of NAFLD-related HCC tumors [11]. Obesity is characterized by a low-grade chronic inflammatory response, which is a risk factor for HCC. Adipose tissue expansion promotes the release of proinflammatory cytokines that can activate pro-oncogenic pathways, such as STAT3 [18], which has been widely reported to promote tumor growth and immune evasion. STAT3 overactivation has been found in different solid tumors, such as bladder, bone, breast, liver, among others [19], correlating with poor prognosis in most of these cancers [20]. Activated STAT3 prevents p53-mediated growth control, thus allowing tumor progression. STAT3 also induces the expression of vascular endothelial growth factor (VEGF), stimulating angiogenesis that favors invasion and metastasis [21]. The canonical inducer of STAT3 activation is IL-6, which is also a master regulator of inflammation and tumor growth [20,22]. In the present work, we aimed at (i) characterizing HCC onset and progression in a background of lipid overload, particularly in cholesterol, and (ii) identifying the clinical relevance of a gene expression signature established from this HCC. Notably, we hypothesized that this gene signature could predict the clinical outcome of patients with HCC by defining a subtype of tumors with high aggressiveness and poor prognosis.

## 2. Materials and Methods

### 2.1. Animals

Sixty-four 14-day-old male mice (strain C57BL/6) were randomly separated into six groups according to Supplementary Table S1. Briefly, these groups include (1) mice fed with regular chow diet (CW); (2) mice fed with chow diet and injected with a single dose of *N*-nitrosodiethylamine (DEN, 10 μg/kg, i.p.) (CW/DEN); (3) mice fed with Western diet (W, catalog #D12089, Research Diets Inc. New Brunswick, NJ, USA); (4) mice fed with Western diet and injected with a single dose of DEN (W/DEN). Later we added two additional groups: (5) mice fed with a high cholesterol diet (HC, 1% cholesterol, #611638, Dyets Inc., Bethlehem, PA, USA); and (6) mice fed with HC diet and injected with a single dose of DEN (HC/DEN). Animals received diet and water ad libitum. After 8 months, mice were euthanized under Avertin (2-2-2 tribromoethanol) anesthesia. Liver weight was documented, liver tissue and serum were collected for subsequent analysis [3]. All animals were maintained in specific pathogen-free housing and cared for in accordance with the NIH Guide for Care and Use of Laboratory Animals and Universidad Autonoma Metropolitana guidelines for the use of animals.

Diet composition, in terms of main components are as follows: CW diet (fat 5%, cholesterol 0.01%, carbohydrates 23.30% and protein 17.10%); Western diet (fat 5%, cholesterol 1.0%, carbohydrates 33.00% and protein 17.50%); and HC diet (fat 5%, cholesterol 1.0%, carbohydrates 23.00% and protein 17.50%).

### 2.2. Histology

Microscopic inspection was evaluated by routine hematoxylin & eosin (H&E) staining. Fibrosis was assessed by Sirius Red staining as previously reported [23]. Steatohepatitis was defined by the presence of steatosis, inflammation and hepatocellular ballooning [24], as per the FLIP algorithm [25]. The severity of steatosis, lobular inflammation and hepatocellular ballooning were scored using the NASH clinical research network (CRN) criteria [26]. Specifically, the amount of steatosis (percentage of hepatocytes containing fat droplets) was scored as 0 (<5%), 1 (5–33%), 2 (>33–66%) and 3 (>66%). Hepatocyte ballooning was classified as 0 (none), 1 (few) or 2 (many cells/prominent ballooning). Foci of lobular inflammation were scored as 0 (no foci), 1 (<2 foci per 200× field), 2 (2–4 foci per 200× field) and 3 (>4 foci per 200× field). The NAFLD activity score (NAS) was computed from the grade of steatosis, inflammation and ballooning [27]. Paraffin sections of human liver tissue (NASH and HCC patients) were generously provided by Dr. Linda Muñoz-Espinosa, University Hospital, UANL.

### 2.3. Cell line Cultures

Huh7, Hep3B, HepG2 and THLE-3 cell lines were obtained from the American Type Culture Collection (ATCC, Manassas, VA, USA), and the PLC-PRF5 cell line was provided by Dr. Jens U. Marquardt. Cells were cultured in William’s medium supplemented with 10% fetal bovine serum (FBS, Hy-Clone, Logan, UT, USA), 100 U/mL ampicillin and 100 µg/mL streptomycin (Thermo Fisher Scientific, Waltham, MA, USA). Cells were maintained at 37 °C in a 5% CO_2_ and 90% humidity atmosphere. Cells were plated in plastic culture bottles (Sigma-Aldrich, Saint Louis, MO, USA). All cell lines were mycoplasma-free. All experiments were carried out by triplicate in the logarithmic phase of growth.

### 2.4. Cholesterol Determination

Total cholesterol was determined as previously reported [28]. Briefly, 10 mg of the liver was saponified with alcoholic KOH in a 60 °C heating block for 30 min. After the mixture had cooled, 3 mL of hexane and 600 μL of distilled water were added and shaken to ensure complete mixing. Appropriate aliquots of the hexane layer were evaporated under nitrogen with a vacuum (Speed Vac, Savant, Cranbury, NJ, USA) and used for cholesterol measurement with O-phthalaldehyde dissolved in acetic acid (0.5 mg/mL) at 550 nm.

### 2.5. Serum Biochemical Determinations

Blood samples were obtained from the retro-orbital sinus under avertin anesthesia. Serum total cholesterol, triglycerides, ALT and AST activities were determined by using an automated analyzer for clinical chemistry (SpotChem EZ, ARKRAY, USA Clinical Diagnostics).

### 2.6. RNA Extraction, Quantification and Integrity

Total RNA was extracted using the QIAGEN RNeasy mini-Kit (Qiagen GMBH, Hilden, Germany) following the manufacturer’s instructions. RNA quantity and purity were estimated using a Nanodrop 2000 Spectrophotometer (Nanodrop Technologies, Wilmington, DE, USA) and integrity was assessed by Agilent 2100 Bioanalyzer (Agilent, Palo Alto, CA, USA).

### 2.7. RNA Sequencing

RNA sequencing was performed using Illumina HiSeq4000 and deposited at the Bio-project database (ID: PRJDB10804). Raw reads were filtered by adapter sequences, contamination, and low-quality reads. The reads were then mapped with mouse genome reference (GRCm38.p6) using HISAT2 (hisat2-2.0.2-beta) followed by read summarization with featureCounts (subread-1.5.0-p1). All data analysis was performed using R programing language and related packages. The output matrix from featureCounts was input into Bioconductor DESeq2 for differential expression analysis with a false discovery rate (FDR) of 0.5, and *p*-value of 0.001 and a log fold change from −1.5 to 1.5. Significance testing was performed using Wald Test statistics. To visualize the data, heatmaps and principal component analysis (PCA) were a performance. All plots were generated using the MADE4 package [29].

### 2.8. Enrichment Analysis

Gene set enrichment analysis (GSEA) was performed using GSEA software provided by Broad Institutes (http://software.broadinstitute.org/gsea/index.jsp (accessed on 15 June 2020)). Gene sets from the MSigDB database were tested, and gene sets with NOM *p*-value < 0.05 and FDR < 0.25 were considered significantly enriched in a priori defined set of genes. We used ingenuity pathway analysis (IPA, Ingenuity Systems, Inc., Redwood City, CA, USA) to investigate the biological pathways and networks associated with model genes using the whole dataset. Genes with their log2-fold change were used as input. We considered a disease, biological functions, and upstream regulators to be significant if the absolute value of their activation *z*-score is > 1.5. The *z*-score reflects the match between the observed gene expression and the predicted relationship direction. It is used to infer activation states of called functions and regulators [29].

### 2.9. Immunofluorescence (IF)

Tissue was either fixed in 4% formaldehyde and preserved in Tissue Tek for cryosections and cut in 3–5 μm sections. Subsequently, these were washed with PBS-tween for 5 min. The specific antibodies (Supplementary Table S3) were diluted in PBS with 0.1% horse serum and incubated overnight at 4 °C in a humid chamber. The tissues were washed again with PBS, incubated with DAPI for 5 min. Finally, slides were examined using the Carl Zeiss LSM 780 NLO confocal microscope (Carl Zeiss, Inc., Jena, Germany).

### 2.10. Quantitative RT–PCR

After DNase digestion with a DNA-free kit (Ambion Inc., Austin, TX, USA), 500 ng (tissue) and 100 ng (cells) total RNA was reverse transcribed in 20 μL reaction volume with a SuperScript first-strand synthesis kit according to the manufacturer’s instructions (Invitrogen/Thermo Fisher Scientific, Waltham, MA, USA). Real-time quantitative PCR analysis was performed with a CFX96 Touch (Bio-Rad, Hercules, CA, USA) thermal cycler in a 96-well reaction plate. The 10 μL PCR reaction mix contained 5 μL 2X SYBR green PCR master mix (Bio-Rad), 200 nM of each primer, and 1 μL cDNA template. Reactions were incubated for 10 min at 95 °C followed by 40 cycles of 30 s at 95 °C and 60 s at specific primer temperature. Melting analysis of the PCR products was also conducted to validate the amplification of the specific product. The expression level of mouse rs18 was used as an internal reference. Relative gene expression levels were calculated with the 2^−∆∆CT^ method [30]. Primer sequences are listed in Supplementary Table S2.

### 2.11. Mouse Serum MultiPlex Measurements

Cytokines and growth factors were measured in mouse sera using the mouse angiogenesis/growth factor magnetic bead panel bead assay (EMD Millipore Corporation, Billerica, MA, USA) and a MAGPIX instrument using Luminex xMAP Technology. The analysis was performed according to the manufacturer’s instructions.

### 2.12. Data Mining

The RNA-seq data level 3 (Counts files) of 371 patients with HCC, and 50 samples of normal liver, were retrieved from The Cancer Genome Atlas (TCGA) database (https://portal.gdc.cancer.gov/ (accessed on 20 November 2020)) until 28 January 2018. Moreover, 101 microarray data from the Laboratory of Experimental Carcinogenesis (LEC/NCI/NIH) was used. The parameters for the differential expression analysis were false discovery rate (FDR) equal to 0.5, *p*-value < 0.001 and log fold change from −1.5 to 1.5. Moreover, clustering analysis was done using Cluster 3.0 and TreeView 1.6 using uncentered correlation and average linkage options. Integration of genomic data was conducted as previously described using publicly available gene expression data sets downloaded from GEO [7].

### 2.13. Survival Analysis

For survival analysis, expression data were categorized using a *z*-score cutoff of 0. Kaplan–Meier method using the log-rank test was used to estimate survival curves [31].

### 2.14. Statistical Analysis

Each animal and cell experiment was performed at least in triplicate using cells of at least ± 3 different passages. Data are reported as the average ± standard error (SEM). Pearson’s correlation analysis was used to assess the correlation between expression of Slc41a3, Fabp5, Igdcc4, mthfd1l and total cholesterol content in human liver cancer cell lines. For the comparison of means of different groups, an analysis of variance (ANOVA) was used, followed by multiple comparisons by the Tukey’s test. The GraphPad Prism software (v. 8.2.1) was used. Differences were considered significant at *p* ≤ 0.05.

## 3. Results

### 3.1. High Lipid Diets Induce Weight Gain, Liver Damage and Tumor Promotion

**Figure 1:**
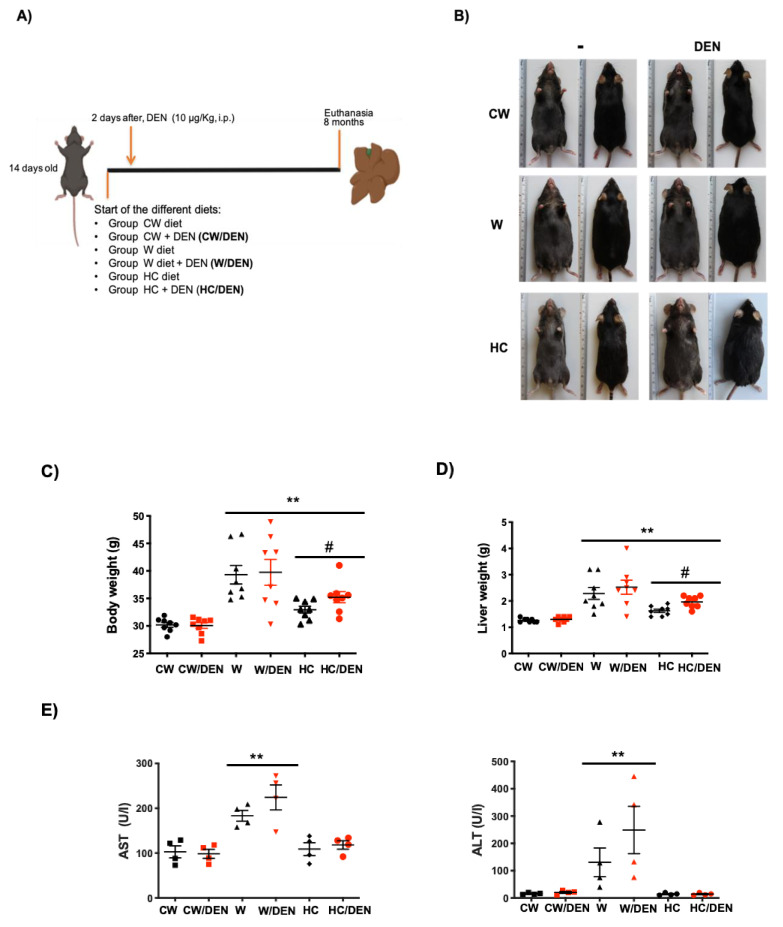
Lipid-enriched diets induce weight gain and liver damage. 14 days-old male mice were fed for 8 months with western and high cholesterol diets as stated in Material and Methods. (**A**) Experimental design. CW, chow standard diet; W, Western diet; HC, high cholesterol; DEN, *N*-nitrosodiethylamine. (**B**) Mice under the W and HC diets presented obesity. Representative images (*n* = 8). (**C**) body weight (*n* = 8) and, (**D**) liver weight (*n* = 8), of mice under different diets. (**E**) Liver damage was addressed by serum activity of aspartate aminotransferase (AST) and alanine aminotransferase (ALT) (*n* = 4). Each graph plots the individual data points, the superimpose horizontal line indicates the arithmetic mean and error bar shows ± SEM. ** *p* < 0.01 vs. CW groups; # *p* < 0.01 vs. W groups.

Mice fed western and cholesterol diets for eight months (Figure 1A) presented body weight gain (Figure 1B,C) as a consequence of increased visceral fat deposition (Supplementary Figure S1A, white arrows), comparing with CW fed animals. Increased liver weight was also observed (Figure 1D), but the liver-to-body weight ratio was unchanged (data not shown) as a result of the obesity observed in animals under this lipid-enriched diet (Supplementary Figure S1A). The phenotype was closely associated with a significant increment in AST and ALT serum activities (Figure 1E) in mice under a Western diet, indicating that hepatocellular damage persists at eight months of treatment. Interestingly, HC/DEN group did not change AST and ALT values at eight months. No significant difference in liver damage tests was observed in animals treated or not with DEN, but changes were remarkable comparing with animals fed with the regular CW diet.

**Figure 2:**
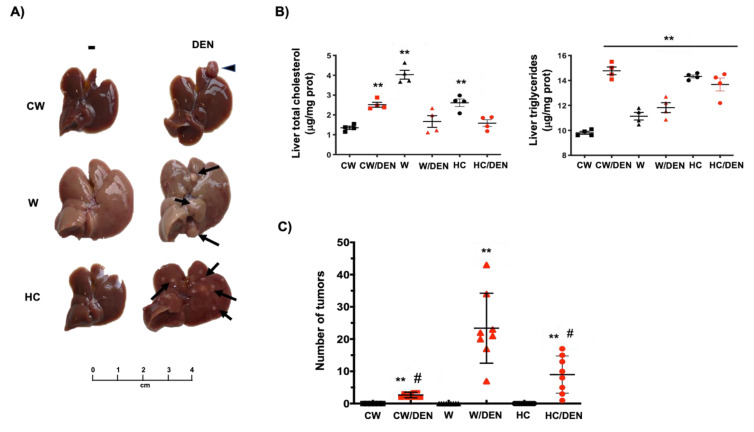

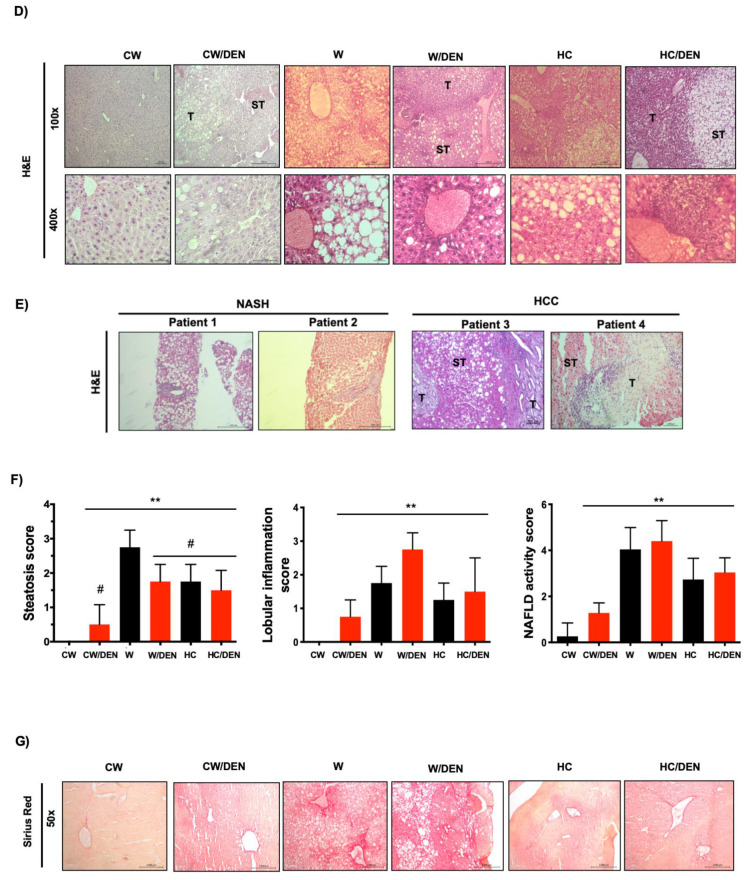
The consumption of a Western diet induces lipid liver overload and hepatic tumors. (**A**) Gross inspection of the livers from mice fed with the different diets and treated or not with DEN. Arrows and arrowheads show tumors. Representative images of at least eight animals per group. (**B**) Liver cholesterol and triglycerides content (*n* = 4). (**C**) Number of tumors in livers from different groups of mice. Each graph plots the individual data points, the superimpose horizontal line indicates the arithmetic mean and error bars showing ± SEM (*n* = 8). (**D**) mouse and (**E**) human liver tissue histology assessed by routine H&E. Each mouse image is representative of four animals. T, tumor; ST, surrounding tissue. (**F**) histological scores. (**G**) Fibrosis determined by Sirius red staining. Each image is representative of four animals. Each bar is the mean ± SEM. ** *p* < 0.01 vs. CW group; # *p* < 0.05 vs. W diet alone group.

In agreement with liver damage, we found the macroscopic changes associated with steatosis, including the characteristic yellowish color in the livers, particularly in those under the Western diet (Figure 2A) comparing with CW-fed animals. Liver lipid overload was biochemically corroborated by testing total cholesterol and triglycerides. Consistently, all experimental groups exhibited increased values of both lipids regarding CW-fed mice (Figure 2B). Next, we proceeded to identified tumors in livers from DEN-treated mice. The number of tumors in W/DEN and HC/DEN mice were higher (23.3 ± 10.3 and 9 ± 1.9 tumors, respectively) than in CW/DEN mice (2.7 ± 0.2 tumors) (Figure 2C), suggesting synchronous development of several independent tumors, which is a well-defined characteristic in HCC [32]. Protruding tumors were observed in W/DEN livers (Figure 2A, arrow), comparing with CW/DEN livers, where some pedunculated tumors were identified (Figure 2A, arrowhead), while HC/DEN animals showed the characteristic HCC protruding tumors. In addition, W/DEN tumors exhibited increased vascularity (Supplementary Figure S1B yellow arrow), a distinctive feature of aggressive HCC [30].

Histological analysis by H&E staining revealed discrete borders and irregular wide trabeculae (>3 cells) in W/DEN tumors. Animals fed with a Western diet presented massive steatosis disrupting normal architecture. Consistently, steatosis (micro and macrovesicular) was most pronounced in surrounding tissue (ST) areas, while lipid content in tumor (T) zones was only marginal and replaced by increased lobular inflammation. The same pattern was also identified in HC/DEN, but the microsteatosis predominated (Figure 2D). Interestingly, liver tissues from NASH and NASH-derived HCC patients exhibited a similar histological presentation in comparison with the animal models (Figure 2E). Quantification of the histological findings revealed significant values of steatosis, inflammation and NAFLD activity score in all animals’ groups comparing with CW control mice (Figure 2F).

Western diet-induced mild fibrosis, particularly in pericentral zones, but animals in the W/DEN group exhibited both pericentral and perisinusoidal zones with bridging fibrosis (Figure 2G) in comparison with HC/DEN and CW/DEN.

### 3.2. Transcriptional Profile of Tumors from Lipid Overloaded Livers

**Figure 3:**
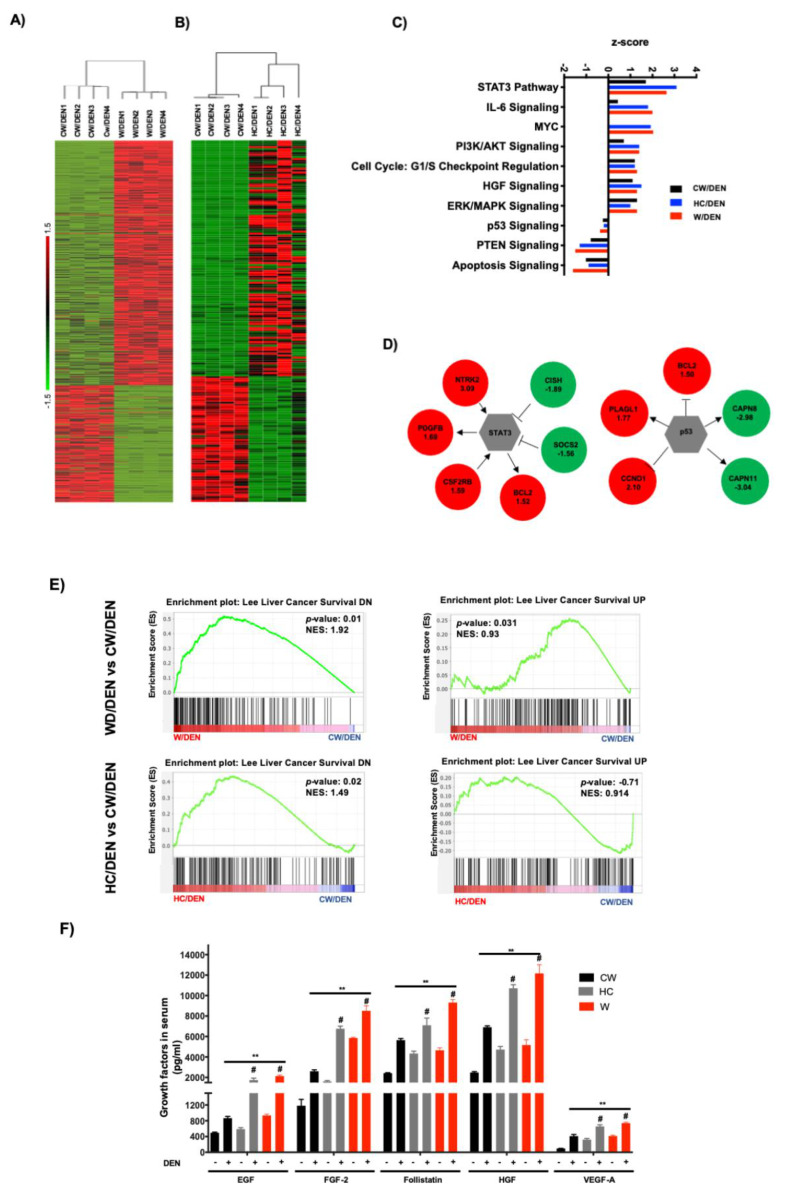

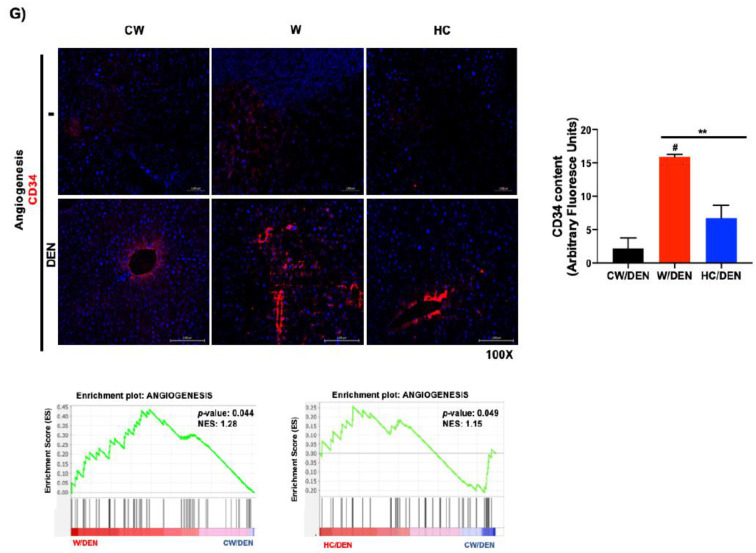
Tumors from lipid-enriched diets exhibit a profound change in gene expression favoring survival and proliferation signaling pathways and decreasing key tumor suppressor routes. Tumor tissues from mice fed with chow diet and injected with a single dose of *N*-nitrosodiethylamine (DEN, 10 μg/kg, i.p.) (CW/DEN), mice fed with Western diet and injected with a single dose of DEN (W/DEN), mice fed with HC diet and injected with a single dose of DEN (HC/DEN) were subjected to RNA-sequencing as stated in Material and Methods. (**A**) Heatmaps showing the good separation of (A) W/DEN tumors vs. CW/DEN and (**B**) HC/DEN tumors vs. CW/DEN red and green colors indicate high and low gene expression, respectively (*n* = 4). (**C**) Process networks analysis in tumors from CW/DEN (black), high cholesterol HC/DEN, (blue) and W/DEN (red) (*n* = 4). The top rank-ordered processes and networks are based on statistical significance (*p* < 0.01). (**D**) Expression targets of Stat3 and p53 in tumor signature. (**E**) Gene set enrichment analysis (GSEA) of liver cancer survival (down and upregulated) data sets comparing tumors from W/DEN and HC/DEN to CW/DEN. (**F**) Growth factors serum concentration of all mice groups. Proteins were determined by based multiplex immunoassay (*n* = 4). Each bar represents the average ± SEM ** *p* < 0.01 vs. CW group; # *p* < 0.05 vs. W diet alone group. (**G**) Angiogenesis was judged by immunofluorescence of CD34, representative images of four animals. Original magnification, 100X. Quantification of CD34 fluorescence and GSEA of angiogenesis, data set comparing W/DEN and HC/DEN to CW/DEN (*n* = 4).

To identify the transcriptional alterations in tumors under western and HC diets consumption, we performed RNA-sequencing. Differentially expressed genes (DEG) between W/DEN and HC/DEN versus CW/DEN group. The analysis highlighted 671 DEG in W/DEN; and 194 DEG in HC/DEN (adjusted *p* value below 0.01 and a threshold of 1.5-fold change). In the case of W/DEN analysis, we found 217 downregulated genes and 454 upregulated genes (Supplementary Table S4), and in HC/DEN, we found 74 genes significantly downregulated, and 120 genes were upregulated when comparing with CW/DEN (Supplementary Table S5). These genes were highly efficient in separating the tumors developed under the lipid-enriched diets versus the regular chow diet (Figure 3A,B). To corroborate RNA-sequencing data, we performed real-time RT–PCR to analyze the expression of eight genes in samples not included in the RNA-seq, as technical and biological validation, respectively (Supplementary Figure S2). For all tested genes, real-time RT–PCR results recapitulated the RNA-seq data.

Ingenuity pathway analysis (IPA, Figure 3C) of the W/DEN and HC/DEN signatures showed that the dominant functional network upregulated was involved in IL-6/Stat3 pathway (Bcl2, Cish, Socs2, Ntrk2, Pdgfb, Csf2rb), cell cycle/Myc (Bcl2, Pak1, Rapgef4, App), PI3K/Akt signaling Bcl2, Bcl2l1, Ccnd1, Cdkn1a). Notably, key tumor-suppressive pathways were downregulated, such as p53 (Bcl2, Bcl2l1, Ccnd1, Cdkn1a), PTEN (Ikbke, Iitga2, Iitga4, Ntrk2), and apoptosis (Bcl2, Bcl2l1, Capn11, Capn8). The involvement of STAT3 and p53 networks (Figure 3D) suggests an aggressive phenotype in tumors generated in a lipid overloaded environment. Supporting this hypothesis, gene set enrichment analysis (GSEA) identified an abundance of genes involved in poor prognosis in animals fed with W and HC diets. Accordingly, Lee liver cancer survival DN gene set was significantly enriched in CW/DEN, whereas liver cancer survival UP [31] gene set was enriched in W/DEN and HC/DEN tumors, indicating the worst prognosis in W/DEN and HC/DEN groups compared to CW/DEN (Figure 3E). To corroborate these findings, we performed an immunoassay (MultiPlex) to address the serum content of different growth factors and cytokines associated with unfavorable prognosis in HCC, such as EGF, FGF-2, Follistatin, HGF and VEGF-A [30,33,34,35]. An increment in the expression of these soluble factors was observed in the serum of W/DEN mice comparing with CW/DEN mice (Figure 3F). Interestingly, most of the increased growth factors are related to survival and angiogenesis. To confirm the relevance of angiogenesis, we assayed the content of CD34, a canonical marker of angiogenesis, highlighted a strong content in W/DEN tumor samples comparing with CW/DEN. No signal was detected in the other groups. Activation of angiogenesis was further corroborated by GSEA, demonstrating a significant enrichment of an angiogenesis-related gene set in the expression profiles of tumors derived from W/DEN mice (Figure 3G).

### 3.3. Inflammation Present in Liver Tumors Associated with Cholesterol Consumption

**Figure 4:**
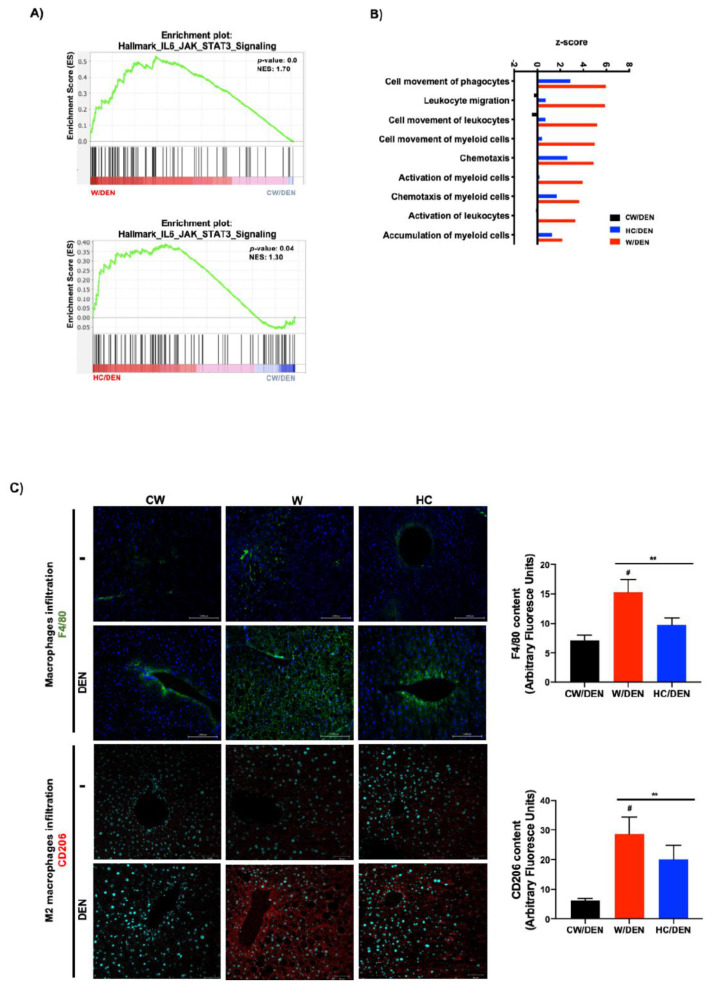

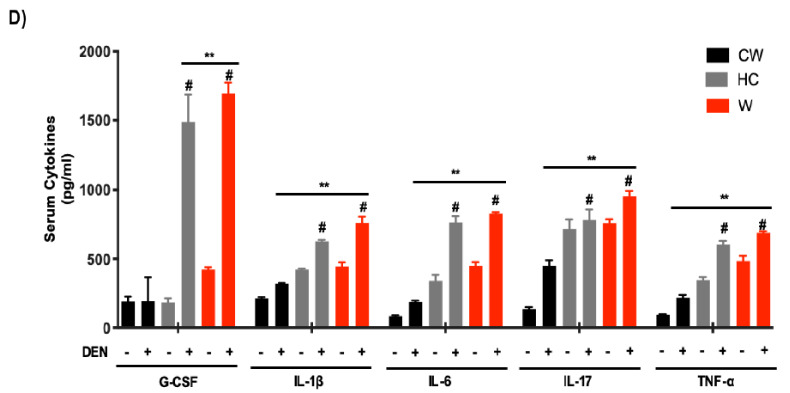
Inflammation is a distinctive feature in liver tumors associated with lipid-enriched and cholesterol diet consumption. (**A**) Gene set enrichment analysis (GSEA) of hallmark IL6-Jak-STAT3 signaling, data set comparing W/DEN and HC/DEN to CW/DEN (*n* = 4). (**B**) Process networks analysis related to inflammation in tumors from CW/DEN (black), W/DEN (red) and HC/DEN (blue) (*n* = 4). The top rank-ordered processes and networks are based on statistical significance (*p* < 0.01). (**C**) Macrophages infiltration by immunofluorescence of F4/80 (green fluorescence) and CD206 (M2 phenotype, red fluorescence), representative images of four animals per group, original magnification, 100X. Quantification of F4/80 and CD206 fluorescence. (**D**) Proinflammatory cytokines serum concentration of all mice groups. Proteins were determined by based multiplex immunoassay (*n* = 4). Each bar represents the average ± SEM ** *p* < 0.01 vs. CW group; # *p* < 0.05 vs. W diet alone group.

To corroborate the involvement of the STAT3-signaling pathway in the phenotype of NAFLD-associated HCC in mice, GSEA was performed using the Hallmark_IL6_JAK_STAT3_Signaling gene set [36]. This specific signature was significantly enriched in the expression profiles of tumors derived from mice under lipid-enriched diets, suggesting an activation of the IL6/STAT3 pathway (Figure 4A). STAT3 is a well-known key driver of inflammation. Accordingly, IPA revealed a clear inflammatory disorder judged by the top signaling pathways activated (Figure 4B). To corroborate this hypothesis, macrophage infiltration was evaluated by both F4/80 and the specific M2-macrophages marker CD206 in mouse tissue from experimental groups (Figure 4C). As expected, a massive M2 macrophages infiltration was observed in tumors developed in mice under steatogenic diets (W or HC diet), comparing with liver tissue under the diets alone (i.e., without DEN) or in CW/DEN group, which presented slight positive staining, particularly in the pericentral zone. To support further the inflammatory phenotype, multiplex analysis of the main proinflammatory mediators demonstrated an increment in the expression of G-CSF, IL6, IL1β, IL17, and TNF-α (Figure 4D).

**Figure 5:**
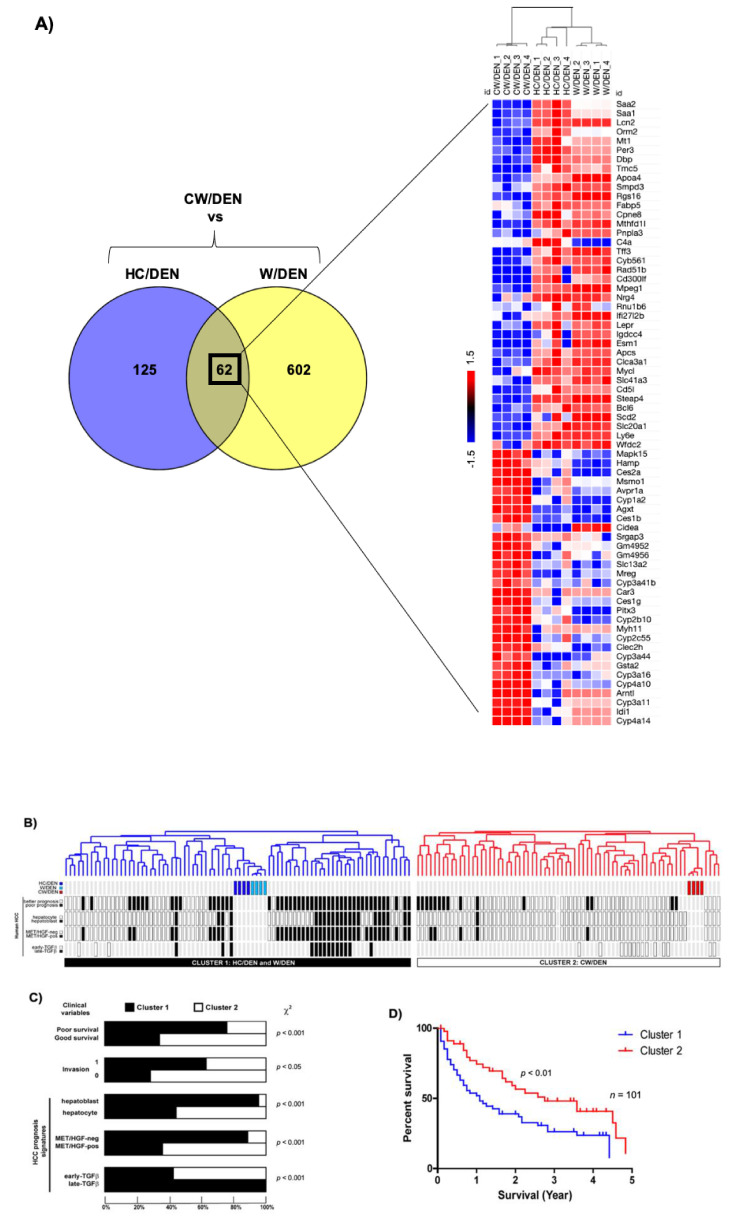
Cholesterol-enriched diet consumption promotes liver tumors. (**A**) Venn diagram differentially depicting expressed common genes between HC/DEN and W/DEN groups (*n* = 4). Heatmap shows the common 62 genes indicating the fold change value. (**B**) Dendrogram overview of mice tumors integrated with 101 cases of human HCC. Clustering analysis was based on the expression of 62 ortholog genes in W/DEN and HC/DEN. Two clusters (1 and 2) were identified. (**C**) Distribution of human HCC samples between previously described subgroups with respect to survival (better vs. poor prognosis), cell origin (hepatoblast vs. hepatocytes), activation of MET/HGF), and TGF-β-signaling pathway (early vs. late) is indicated on the left. F, statistical analysis of HCC distribution between clusters 1 and 2 based on previous gene signatures and clinical parameters. Cluster 1, which is defined by the W/DEN and HC/DEN signature, shows a significant enrichment in HCCs with the following features: bad survival, hepatoblast traits, activation of MET/HGF, and late TGF-β pathways. (**D**) Kaplan–Meier plots and log-rank statistics analysis revealed a significant decrease in overall survival for patients included in cluster 1.

Integrative genomics was performed to evaluate the clinical relevance of the crosstalk between mouse HCC and human HCC. The cholesterol signature (62 genes shared by W/DEN and HC/DEN) shared by W/DEN and HC/DEN groups in comparison with CW/DEN (Figure 5A) was first integrated with the gene expression profile of 101 cases of human HCCs from the LEC cohort, which were extensively characterized [37]. Hierarchical clustering analysis of the integrated data set identified 2 robust clusters whose: cluster 1, W/DEN and HC/DEN tumors, cluster 2 CW/DEN tumors (Figure 5B). Strikingly, cluster 1 included significant tumors, which were assigned to a poor prognosis group than cluster 2 (Figure 5C,D). As shown in Figure 5C, cluster 1 was defined by poor survival [31], hepatoblast traits, along with the activation of MET/HGF [30] and late TGF-β signaling [38]. In addition, we identified signs of vascular invasion in cluster 1. The percent survival of patients in cluster 1 was significantly reduced (*p* < 0.01) in comparison with cluster 2 (Figure 5D).

The analysis of the 62 orthologous genes in the human TCGA database identified 4 genes differentially overexpressed related to high lipid overload with potential relevance in tumor promotion: Mthfd1l, (W/DEN: 2.74- and HC/DEN: 2.42-fold change) Slc41a3, (W/DEN: 2.67- and HC/DEN: 1.70-fold change), Fabp5 (W/DEN: 2.20- and HC/DEN: 2.70-fold change); and Igdcc4, (W/DEN: 2.05- and HC/DEN: 1.88-fold change increased) in W/DEN and HC/DEN, respectively comparing with CW/DEN. We verified by qRT–PCR the expression of these genes in mouse samples ( Supplementary Figure S3).

**Figure 6:**
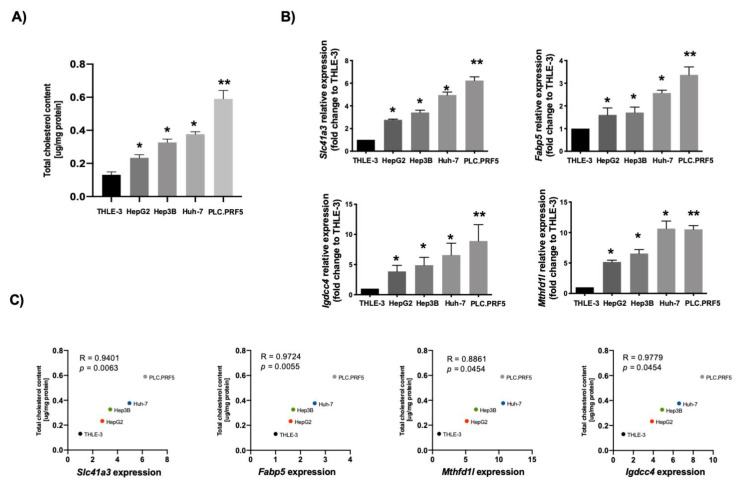
Lipid-associated four-gene signature of hepatocellular carcinoma (HCC) correlates with cholesterol overload in human cancer cell lines. (**A**) Total cholesterol quantification in different human HCC-derived cell lines (*n* = 4). (**B**) Slc41a3, Fabp5, Igdcc4 and Mthfd1l gene expression addressed by qRT–PCR in different HCC-derived human cell lines. THLE-3 cells were used as control (*n* = 4). Each bar represents the average ± SEM * *p* < 0.05; ** *p* < 0.01 vs. THLE-3 cells. (**C**) Pearson’s correlation analysis between total cholesterol content and gene expression of Slc41a3, Fabp5, Igdcc4 and Mthfd1l (*n* = 4).

Then, we explored the correlation between the expression of these genes and cholesterol content in four well-known human HCC cell lines using, as a control, the noncancerous hepatocyte-like THLE-3 cell line. Figure 6A depicts the differences in cholesterol content in the different cell lines. We determined the expression of the four genes in those cell lines, and a significant correlation between gene expression and cholesterol content was observed for all genes examined (Figure 6B,C).

**Figure 7:**
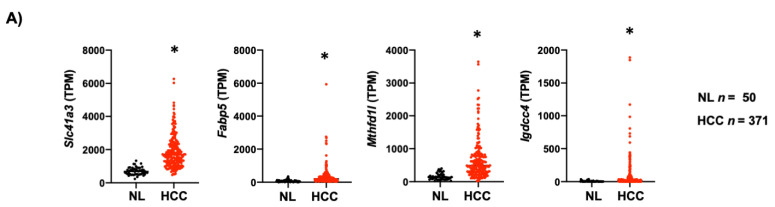

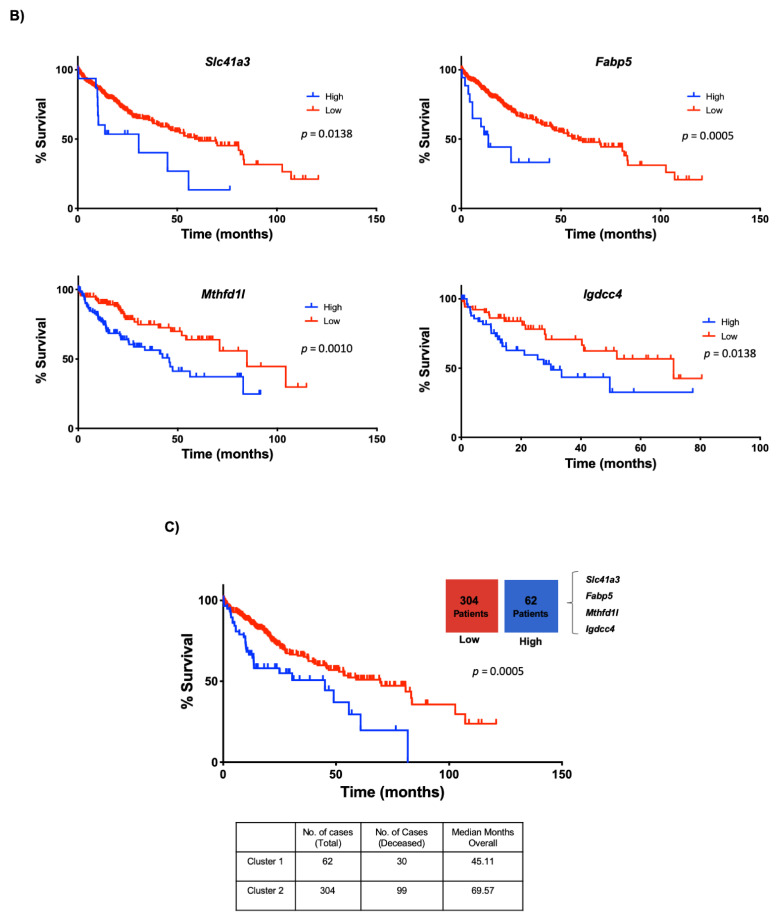
Lipid-associated four-gene signature defines a subtype of human liver cancer with a poor prognosis. (**A**) Plots chart showing higher expression of Slc41a3, Fabp5, Igdcc4 and Mthfd1l in human HCC (*n* = 371) compared to no-tumor liver tissue (NL, *n* = 50) according to the TCGA database (https://www.cbioportal.org/, accessed on 10 November 2020). TPM, transcripts per million. * *p* < 0.01 vs. normal liver (NL). (**B**) Kaplan–Meier curves of overall survival in HCC patients. Patients with HCC were separated into two groups, high (red) and low (black) expression of Slc41a3 (*p* = 0.0138), Fabp5 (*p* = 0.0005), Igdcc4 (*p* = 0.0010), and Mthfd1l (*p* = 0.0328). (**C**) HCC data set from the TCGA was divided into unaltered and altered groups regarding the four-gene signature, and the overall survival in HCC patients was addressed and plotted in a Kaplan–Meier curve (altered red group) and low (unaltered black group).

The clinical relevance of mouse data in human liver cancer was determined by analyzing the expression of the four genes in the TCGA dataset, including 50 non-tumor liver tissue (NL) and 371 HCC samples (LIHC, Liver Hepatocellular Carcinoma). The four-gene signature associated with mouse NASH was increased in human HCC (Figure 7A). Next, we evaluated the capacity of these genes to predict survival in the 371 human HCC. For all genes analyzed, high expression was associated with a poor outcome (Figure 7B). Finally, we grouped the four genes and performed the analysis between patients with altered (*n* = 62) vs. unaltered (*n* = 304) gene expression. As expected, Kaplan–Meier plot and log-rank testing demonstrated a reduced overall survival for patients included in the altered gene group (*p* < 0.001) (Figure 7C). Thus, the molecular differences between these two subclasses of HCC were associated with a remarkable difference in the clinical outcome.

## 4. Discussion

Previously, we demonstrated the liver tumor promotion properties elicited by the consumption of a cholesterol-enriched diet in mice. Tumors presented an aggressive phenotype, including lung metastasis, comparing with animals fed a regular chow diet [3]. This outcome was strongly associated with reactive oxygen species (ROS)-induced DNA damage, failure in the activation of the major DNA repair machinery and apoptosis resistance. The main objectives of the present work were to characterize the HCC progression in NASH induced by the consumption of lipid-enriched diets, particularly the contribution of cholesterol and, to identify the gene expression profile associated with this particular feature. Thus, we fed animals up to 8 months with a commercial and well-studied Western diet (high lipid, cholesterol and carbohydrates). Then, mice received a single dose of DEN to induce liver carcinogenesis, as we previously reported [3,39].

Under the Western diet, mice exhibited obesity, body weight gain due to the accumulation of visceral fat, and the presence of liver damage. These findings are in agreement with those observed in humans under an occidental (western) pattern diet [10] and in other well-characterized mouse models of NASH [27,40]. The cotreatment with DEN efficiently induced a higher burden of HCC comparing with CW/DEN groups, clearly suggesting that lipid overload in the liver acts as a tumor promoter. One of the key components of the Western diet is high cholesterol. We and others have published that this lipid, in addition to the well-recognized fundamental properties for life, displays noxious effects when it is excessively accumulated in cells, particularly as a result of mitochondrial dysfunction and impairment of signal transduction through altering cellular membrane fluidity [12,23,28]. We previously demonstrated that cholesterol definitively increases tumor burden and aggressivity [3,14]. However, the exact molecular mechanism by which cholesterol promotes tumor development is not fully understood.

The transcriptomic analysis carried out by RNA-seq shows a good separation between CW/DEN and lipid-enriched diets (W/DEN, HC/DEN) groups (Figure 3A,B). GSEA revealed a noticeable inflammatory phenotype, particularly associated with IL-6/STAT3 signaling, in addition to other pathways related to growth factors, such as HGF, including PI3K/AKT and ERK pathways. It was interesting the abrogation of key tumor-suppressive signals, such as p53, PTEN and apoptosis signaling observed in the model (Figure 3C). Our previous work identified a remarkable mechanistic effect induced by cholesterol overload in the liver, including downregulation of the main DNA repair proteins, such as ATM, ATR, CHK1 and 2, and p53 [3]. In the present work, IPA confirmed this effect in terms of signaling pathways controlled by p53. Even more, interesting crosstalk between p53- and STAT3-signaling pathways, by the intervention of PI3K and MAPK signaling, has been recently reported in ovarian cancer [41] and linked to an aggressive phenotype mediated by the epithelial–mesenchymal transition, resulting in cell metastasis and chemoresistance.

Since the early 2000s, some reports pointed out the transcriptional repression of p53 by STAT3 [42]. p53 loss of function has also been associated with STAT3 activation in pancreatic cancer [43]. We previously reported that cholesterol overload in the liver, due to consumption of cholesterol-enriched diet for 30 days, presented a remarkable activation of STAT3 and the increment in the expression of key target genes, such as Mcl-1, these were associated with apoptosis resistance [28]. It is clear that cholesterol controls these relevant signaling pathways in preparation for tumor promotion from the beginning of the process. In contrast, it has been observed that dephosphorylation of STAT3 could stabilize p53 and leads to apoptosis [44].

The modulation of the immune response that promotes macrophage recruitment and activation has been previously reported in tumors reared under the consumption of a cholesterol-enriched diet [45]. The close contact of macrophages and tumor cells is associated with M2-like macrophage polarization increasing the aggressiveness of the tumors [46]. In agreement with these previous findings, we observed prominent F4/80 (canonical macrophage marker) and CD206 (M2-macrophage marker) signals in tissues from mice fed lipid-enriched diets, particularly in the Western diet (Figure 5C). Interestingly, it has been observed that M2-macrophages could provide cholesterol to tumor cells, making a cholesterol codependence between these cells in the tumor microenvironment [47]. Recently, Bao and collaborators reported that a high M2-macrophages infiltration is associated with poorer survival and an increase in cancer recurrence [48].

In addition to well-defined macrophage infiltration, we also observed an increase in pro-angiogenic response, judged by the increment in VEGF-A and HGF content and confirmed by CD34 IF, supporting the aggressive phenotype. Accordingly, we reported that HGF/Met-regulated expression signature was associated with an increment in angiogenesis and was able to define a subset of human HCC with poor prognosis and aggressive phenotype [30]. Another characteristic observed in this signature was the prominent decompensation of lipogenesis and oxidative stress.

To gain more mechanistic evidence of the relevance of tumor development in the liver with lipid overload, we studied another group of mice fed with a high cholesterol diet (carbohydrates and other lipids in standard contents). Gene expression analysis in this tumorigenic process identified 194 differentially expressed genes relating to CW/DEN. Comparison between HC/DEN vs. W/DEN identified 62 commonly regulated genes (Figure 5A). Supporting these findings, we established integrative genomics between tumors from mice fed cholesterol-enriched diets and human HCC, where these 62 genes shared by W/DEN and HC/DEN had clinical relevance and are associated with poor prognosis in human HCC. Further research is required to fully establish the contribution of each specific gene, including four outstanding genes (*Slc41a3, Fabp5, Mthfd1l* and *Igdcc4*) with clinical relevance in human cancer. These genes are elevated in HCC samples and are associated with poor survival. Clustering these four genes and separating human HCC in those with altered expression (*n* = 62) regarding patients with unaltered expression (*n*= 304) observed a significant decrement in survival (Figure 7C).

The solute carrier (Slc) family 41 comprises three members, including the Slc41a3. It is related to cation transmembrane transporter activity, driving the efflux of Mg^2+,^ particularly in mitochondria rather than plasma membrane [49]. Slc41a3 overexpression has been reported in cancer for nutrient and Mg^2+^ transport to meet the needs for aberrant metabolism and proliferation [50]. As we have mentioned, mitochondria are the main target of excess cholesterol. Overexpression of this transporter system could be directed to compensate for the loss of fluidity in the mitochondria, improving the extrusion of Mg^2+^.

*Fabp5* encodes for the fatty acid-binding protein 5 (Fabp5). This protein has been related to malignant potential in several cancers, including HCC. Positive staining of Fabp5 in HCC is associated with poor prognosis, recurrence, metastasis and vascular invasion [51]. Fabp5 transports lipids intracellularly for storage purposes, providing building blocks for membrane construction and energy supply, both highly required in the proliferating cells [52].

*Igdcc4* encodes the neighbor of Punc E11, also known as Nope. It is a plasma membrane oncofetal stem/progenitor cell marker initially identified in the murine fetal liver [53]. It is barely detected in the adult liver. It has been previously showing that Nope is a sensitive marker of HCC [54] associated with stemness resembling early postnatal hepatocytes. Although Nope is scarcely detected in adult hepatocytes, the damage could induce the expression, particularly in the cholestatic damage, which is associated with changes in hepatocyte polarization [53]. The Nope protein has been demonstrated to be a good and confident marker for HCC in the clinic because it could be highly detected in AFP-positive and -negative tumors [54].

The methylenetetrahydrofolate dehydrogenase 1 like (Mthfd1l), encoded by the *Mthfd1l* gene, is an enzyme of the folate cycle. It is involved in formate generation, therefore, in the metabolism of mono carbon compound fundamental in both purine synthesis and protein synthesis initiation. Consequently, this enzyme is highly relevant in proliferating tissues, particularly in cancer [55,56]. *Mthfd1l* expression has been associated with poor prognosis, notably in HCC [57]. In normal conditions, MTHFD1L is expressed in mitochondria in embryonic tissues [58]; once again, the mitochondria as an important target in our model. Interestingly, MTHFD1L also has been associated with redox homeostasis in cancer and, as we have demonstrated, one of the main consequences in cancer development in the liver with lipid overloaded is the disruption of redox balance [3]. Metabolic reprogramming is one of the key features of cancer. The proposed four-gene signature clearly indicates a metabolic adaptation, compromising mitochondrial adaptation and macrophage participation, particularly the M2-like polarization in a process induced and driven by cholesterol overload.

## 5. Conclusions

In conclusion, we have characterized an animal model of NASH-derived HCC that recapitulates the human disease, with a clear inflammatory and angiogenic component. RNA-seq analysis identified a specific gene expression profile defining a four-gene signature with potential prognostic applications in the clinical practice, predicting poor survival in patients with HCC. Dietary interventions controlling the quantity and, even more important, the quality of dietary lipids could decrease the incidence of aggressive liver cancers.

## Data Availability

Data available on request from the authors.

## Supplementary Materials

The following are available online at https://www.mdpi.com/article/10.3390/cancers13071721/s1, Figure S1: (A) Gross inspection of mice fed with the different diets and treated or not with DEN. Representative image of at least eight animals, white arrows show adipose tissue. (B) Tumor gross inspection of mice under different diets. Representative image of at least eight livers, yellow arrows show vascularity. Images are representative of at least eight mice. Figure S2: Expression of eight randomly selected genes in samples not included in the RNA-seq, as technical and biological validation. Each graph plots the individual data points, the superimpose horizontal line indicates the arithmetic mean and error bars showing ± SEM. ** *p* < 0.01 vs. CW group; & *p* < 0.05 vs. diet control. Figure S3. Expression of the 4 genes differentially overexpressed related to high lipid overload with potential relevance in tumor promotion: Mthfd1, Slc41a3, Fabp5, Igdcc4. Each graph plots the individual data points, the superimpose horizontal line indicates the arithmetic mean and error bars showing ± SEM. ** *p* < 0.01 vs. CW group.Table S1: Mice groups under different diets and treatments. Table S2: Primers used in the study. Table S3: Antibodies used in the study. Table S4: Gene expression profile of W/DEN vs. CW/DEN. Table S5: Gene expression profile of HC/DEN vs. CW/DEN. Table S6: Gene expression profile shared by W/DEN vs. HC/DEN.

## References

[1] Baffy G., Brunt E.M., Caldwell S.H. (2012). Hepatocellular carcinoma in non-alcoholic fatty liver disease: An emerging menace. J. Hepatol..

[2] Younossi Z.M., Koenig A.B., Abdelatif D., Fazel Y., Henry L., Wymer M. (2016). Global epidemiology of nonalcoholic fatty liver disease-Meta-analytic assessment of prevalence, incidence, and outcomes. Hepatology.

[3] Enriquez-Cortina C., Bello-Monroy O., Rosales-Cruz P., Souza V., Miranda R.U., Toledo-Perez R., Luna-Lopez A., Simoni-Nieves A., Hernandez-Pando R., Gutierrez-Ruiz M.C. (2017). Cholesterol overload in the liver aggravates oxidative stress-mediated DNA damage and accelerates hepatocarcinogenesis. Oncotarget.

[4] Calvisi D.F., Wang C., Ho C., Ladu S., Lee S.A., Mattu S., Destefanis G., Delogu S., Zimmermann A., Ericsson J. (2011). Increased lipogenesis, induced by AKT-mTORC1-RPS6 signaling, promotes development of human hepatocellular carcinoma. Gastroenterology.

[5] Huang D.Q., El-Serag H.B., Loomba R. (2020). Global epidemiology of NAFLD-related HCC: Trends, predictions, risk factors and prevention. Nat. Rev. Gastroenterol. Hepatol..

[6] Cronin K.A., Lake A.J., Scott S., Sherman R.L., Noone A.M., Howlader N., Henley S.J., Anderson R.N., Firth A.U., Ma J. (2018). Annual Report to the Nation on the Status of Cancer, part I: National cancer statistics. Cancer.

[7] Lee J.S., Heo J., Libbrecht L., Chu I.S., Kaposi-Novak P., Calvisi D.F., Mikaelyan A., Roberts L.R., Demetris A.J., Sun Z. (2006). A novel prognostic subtype of human hepatocellular carcinoma derived from hepatic progenitor cells. Nat. Med..

[8] Younes R., Bugianesi E. (2018). Should we undertake surveillance for HCC in patients with NAFLD?. J. Hepatol..

[9] Bhaskaran K., Douglas I., Forbes H., dos-Santos-Silva I., Leon D.A., Smeeth L. (2014). Body-mass index and risk of 22 specific cancers: A population-based cohort study of 5.24 million UK adults. Lancet.

[10] Sun B., Karin M. (2012). Obesity, inflammation, and liver cancer. J. Hepatol..

[11] Massoud O., Charlton M. (2018). Nonalcoholic Fatty Liver Disease/Nonalcoholic Steatohepatitis and Hepatocellular Carcinoma. Clin. Liver Dis..

[12] Mari M., Caballero F., Colell A., Morales A., Caballeria J., Fernandez A., Enrich C., Fernandez-Checa J.C., Garcia-Ruiz C. (2006). Mitochondrial free cholesterol loading sensitizes to TNF- and Fas-mediated steatohepatitis. Cell Metab..

[13] Liu D., Wong C.C., Fu L., Chen H., Zhao L., Li C., Zhou Y., Zhang Y., Xu W., Yang Y. (2018). Squalene epoxidase drives NAFLD-induced hepatocellular carcinoma and is a pharmaceutical target. Sci. Transl. Med..

[14] Garcia-Ruiz C., de la Rosa L.C., Ribas V., Fernandez-Checa J.C. (2020). Mitochondrial Cholesterol and Cancer. Semin. Cancer Biol..

[15] Starley B.Q., Calcagno C.J., Harrison S.A. (2010). Nonalcoholic fatty liver disease and hepatocellular carcinoma: A weighty connection. Hepatology.

[16] Xu F., Rychnovsky S.D., Belani J.D., Hobbs H.H., Cohen J.C., Rawson R.B. (2005). Dual roles for cholesterol in mammalian cells. Proc. Natl. Acad. Sci. USA.

[17] Maxfield F.R., van Meer G. (2010). Cholesterol, the central lipid of mammalian cells. Curr. Opin. Cell Biol..

[18] Stickel F., Hellerbrand C. (2010). Non-alcoholic fatty liver disease as a risk factor for hepatocellular carcinoma: Mechanisms and implications. Gut.

[19] Lee C., Cheung S.T. (2019). STAT3: An Emerging Therapeutic Target for Hepatocellular Carcinoma. Cancers.

[20] Wang Y., Shen Y., Wang S., Shen Q., Zhou X. (2018). The role of STAT3 in leading the crosstalk between human cancers and the immune system. Cancer Lett..

[21] Luo Y., He J., Tao X., Wang H., Fang Q., Guo S., Song C. (2018). miR20b negatively regulates VEGF expression by targeting STAT3 in H22 hepatocellular carcinoma cells. Oncol. Rep..

[22] Hodge D.R., Hurt E.M., Farrar W.L. (2005). The role of IL-6 and STAT3 in inflammation and cancer. Eur. J. Cancer.

[23] Gomez-Quiroz L.E., Seo D., Lee Y.H., Kitade M., Gaiser T., Gillen M., Lee S.B., Gutierrez-Ruiz M.C., Conner E.A., Factor V.M. (2016). Loss of c-Met signaling sensitizes hepatocytes to lipotoxicity and induces cholestatic liver damage by aggravating oxidative stress. Toxicology.

[24] Sanches S.C., Ramalho L.N., Augusto M.J., da Silva D.M., Ramalho F.S. (2015). Nonalcoholic Steatohepatitis: A Search for Factual Animal Models. BioMed Res. Int..

[25] Imhof B.A., Aurrand-Lions M. (2006). Angiogenesis and inflammation face off. Nat. Med..

[26] Lee L., Alloosh M., Saxena R., Van Alstine W., Watkins B.A., Klaunig J.E., Sturek M., Chalasani N. (2009). Nutritional model of steatohepatitis and metabolic syndrome in the Ossabaw miniature swine. Hepatology.

[27] Asgharpour A., Cazanave S.C., Pacana T., Seneshaw M., Vincent R., Banini B.A., Kumar D.P., Daita K., Min H.K., Mirshahi F. (2016). A diet-induced animal model of non-alcoholic fatty liver disease and hepatocellular cancer. J. Hepatol..

[28] Dominguez-Perez M., Simoni-Nieves A., Rosales P., Nuno-Lambarri N., Rosas-Lemus M., Souza V., Miranda R.U., Bucio L., Uribe Carvajal S., Marquardt J.U. (2019). Cholesterol burden in the liver induces mitochondrial dynamic changes and resistance to apoptosis. J. Cell. Physiol..

[29] Castven D., Becker D., Czauderna C., Wilhelm D., Andersen J.B., Strand S., Hartmann M., Heilmann-Heimbach S., Roth W., Hartmann N. (2019). Application of patient-derived liver cancer cells for phenotypic characterization and therapeutic target identification. Int. J. Cancer.

[30] Kaposi-Novak P., Lee J.S., Gomez-Quiroz L., Coulouarn C., Factor V.M., Thorgeirsson S.S. (2006). Met-regulated expression signature defines a subset of human hepatocellular carcinomas with poor prognosis and aggressive phenotype. J. Clin. Investig..

[31] Lee J.S., Chu I.S., Heo J., Calvisi D.F., Sun Z., Roskams T., Durnez A., Demetris A.J., Thorgeirsson S.S. (2004). Classification and prediction of survival in hepatocellular carcinoma by gene expression profiling. Hepatology.

[32] Schlageter M., Terracciano L.M., D’Angelo S., Sorrentino P. (2014). Histopathology of hepatocellular carcinoma. World J. Gastroenterol..

[33] Liu Z., Chen D., Ning F., Du J., Wang H. (2018). EGF is highly expressed in hepatocellular carcinoma (HCC) and promotes motility of HCC cells via fibronectin. J. Cell. Biochem..

[34] Coleman S.J., Grose R.P., Kocher H.M. (2014). Fibroblast growth factor family as a potential target in the treatment of hepatocellular carcinoma. J. Hepatocell. Carcinoma.

[35] Tomoda T., Nouso K., Miyahara K., Kobayashi S., Kinugasa H., Toyosawa J., Hagihara H., Kuwaki K., Onishi H., Nakamura S. (2013). Prognotic impact of serum follistatin in patients with hepatocellular carcinoma. J. Gastroenterol. Hepatol..

[36] Rebouissou S., Amessou M., Couchy G., Poussin K., Imbeaud S., Pilati C., Izard T., Balabaud C., Bioulac-Sage P., Zucman-Rossi J. (2009). Frequent in-frame somatic deletions activate gp130 in inflammatory hepatocellular tumours. Nature.

[37] Lee J.S., Chu I.S., Mikaelyan A., Calvisi D.F., Heo J., Reddy J.K., Thorgeirsson S.S. (2004). Application of comparative functional genomics to identify best-fit mouse models to study human cancer. Nat. Genet..

[38] Coulouarn C., Gomez-Quiroz L.E., Lee J.S., Kaposi-Novak P., Conner E.A., Goldina T.A., Onishchenko G.E., Factor V.M., Thorgeirsson S.S. (2006). Oncogene-specific gene expression signatures at preneoplastic stage in mice define distinct mechanisms of hepatocarcinogenesis. Hepatology.

[39] Takami T., Kaposi-Novak P., Uchida K., Gomez-Quiroz L.E., Conner E.A., Factor V.M., Thorgeirsson S.S. (2007). Loss of hepatocyte growth factor/c-Met signaling pathway accelerates early stages of N-nitrosodiethylamine induced hepatocarcinogenesis. Cancer Res..

[40] Tsuchida T., Lee Y.A., Fujiwara N., Ybanez M., Allen B., Martins S., Fiel M.I., Goossens N., Chou H.I., Hoshida Y. (2018). A simple diet- and chemical-induced murine NASH model with rapid progression of steatohepatitis, fibrosis and liver cancer. J. Hepatol..

[41] Liang F., Ren C., Wang J., Wang S., Yang L., Han X., Chen Y., Tong G., Yang G. (2019). The crosstalk between STAT3 and p53/RAS signaling controls cancer cell metastasis and cisplatin resistance via the Slug/MAPK/PI3K/AKT-mediated regulation of EMT and autophagy. Oncogenesis.

[42] Niu G., Wright K.L., Ma Y., Wright G.M., Huang M., Irby R., Briggs J., Karras J., Cress W.D., Pardoll D. (2005). Role of Stat3 in regulating p53 expression and function. Mol. Cell. Biol..

[43] Wormann S.M., Song L., Ai J., Diakopoulos K.N., Kurkowski M.U., Gorgulu K., Ruess D., Campbell A., Doglioni C., Jodrell D. (2016). Loss of P53 Function Activates JAK2-STAT3 Signaling to Promote Pancreatic Tumor Growth, Stroma Modification, and Gemcitabine Resistance in Mice and Is Associated with Patient Survival. Gastroenterology.

[44] Sainz-Perez A., Gary-Gouy H., Gaudin F., Maarof G., Marfaing-Koka A., de Revel T., Dalloul A. (2008). IL-24 induces apoptosis of chronic lymphocytic leukemia B cells engaged into the cell cycle through dephosphorylation of STAT3 and stabilization of p53 expression. J. Immunol..

[45] Shono S., Habu Y., Nakashima M., Sato A., Nakashima H., Miyazaki H., Kinoshita M., Tsumatori G., Shinomiya N., Seki S. (2011). The immunologic outcome of enhanced function of mouse liver lymphocytes and Kupffer cells by high-fat and high-cholesterol diet. Shock.

[46] Yang Y., Ye Y.C., Chen Y., Zhao J.L., Gao C.C., Han H., Liu W.C., Qin H.Y. (2018). Crosstalk between hepatic tumor cells and macrophages via Wnt/beta-catenin signaling promotes M2-like macrophage polarization and reinforces tumor malignant behaviors. Cell Death Dis..

[47] Goossens P., Rodriguez-Vita J., Etzerodt A., Masse M., Rastoin O., Gouirand V., Ulas T., Papantonopoulou O., Van Eck M., Auphan-Anezin N. (2019). Membrane Cholesterol Efflux Drives Tumor-Associated Macrophage Reprogramming and Tumor Progression. Cell Metab..

[48] Bao D., Zhao J., Zhou X., Yang Q., Chen Y., Zhu J., Yuan P., Yang J., Qin T., Wan S. (2019). Mitochondrial fission-induced mtDNA stress promotes tumor-associated macrophage infiltration and HCC progression. Oncogene.

[49] Mastrototaro L., Smorodchenko A., Aschenbach J.R., Kolisek M., Sponder G. (2016). Solute carrier 41A3 encodes for a mitochondrial Mg^2+^ efflux system. Sci. Rep..

[50] Trapani V., Wolf F.I. (2019). Dysregulation of Mg^2+^ homeostasis contributes to acquisition of cancer hallmarks. Cell Calcium.

[51] Ohata T., Yokoo H., Kamiyama T., Fukai M., Aiyama T., Hatanaka Y., Hatanaka K., Wakayama K., Orimo T., Kakisaka T. (2017). Fatty acid-binding protein 5 function in hepatocellular carcinoma through induction of epithelial-mesenchymal transition. Cancer Med..

[52] Furuhashi M., Hotamisligil G.S. (2008). Fatty acid-binding proteins: Role in metabolic diseases and potential as drug targets. Nat. Rev. Drug Discov..

[53] Bowe A., Zweerink S., Muck V., Kondylis V., Schulte S., Goeser T., Nierhoff D. (2018). Depolarized Hepatocytes Express the Stem/Progenitor Cell Marker Neighbor of Punc E11 After Bile Duct Ligation in Mice. J. Histochem. Cytochem..

[54] Marquardt J.U., Quasdorff M., Varnholt H., Curth H.M., Mesghenna S., Protzer U., Goeser T., Nierhoff D. (2011). Neighbor of Punc E11, a novel oncofetal marker for hepatocellular carcinoma. Int. J. Cancer.

[55] He Z., Wang X., Zhang H., Liang B., Zhang J., Zhang Z., Yang Y. (2020). High expression of folate cycle enzyme MTHFD1L correlates with poor prognosis and increased proliferation and migration in colorectal cancer. J. Cancer.

[56] Agarwal S., Behring M., Hale K., Al Diffalha S., Wang K., Manne U., Varambally S. (2019). MTHFD1L, A Folate Cycle Enzyme, Is Involved in Progression of Colorectal Cancer. Transl. Oncol..

[57] Lee D., Xu I.M., Chiu D.K., Lai R.K., Tse A.P., Lan Li L., Law C.T., Tsang F.H., Wei L.L., Chan C.Y. (2017). Folate cycle enzyme MTHFD1L confers metabolic advantages in hepatocellular carcinoma. J. Clin. Investig..

[58] Pike S.T., Rajendra R., Artzt K., Appling D.R. (2010). Mitochondrial C1-tetrahydrofolate synthase (MTHFD1L) supports the flow of mitochondrial one-carbon units into the methyl cycle in embryos. J. Biol. Chem..

