# Supplementary material for: The Consumption of Cholesterol-Enriched Diets Conditions the Development of a Subtype of HCC with High Aggressiveness and Poor Prognosis
Source: Cancers (Basel). 2021 Apr 6;13(7):1721. doi: 10.3390/cancers13071721 (PMC8038696; doi:10.3390/cancers13071721)
Supplement: Supplementary file 1 [file cancers-13-01721-s001.pdf]

cancers

MDPI

The Consumption of Cholesterol-Enriched Diets Conditions the Development of a Subtype of HCC with High Aggressiveness and Poor Prognosis

Arturo Simoni-Nieves, Soraya Salas-Silva, Lisette Chávez-Rodríguez, Alejandro Escobedo-Calvario, Matthis Desoteux, Leticia Bucio, Verónica Souza, Roxana U. Miranda-Labra, Linda E. Muñoz-Espinosa, Cédric Coulouarn, María Concepción Gutiérrez-Ruiz, Jens U. Marquardt and Luis E. Gomez-Quiroz

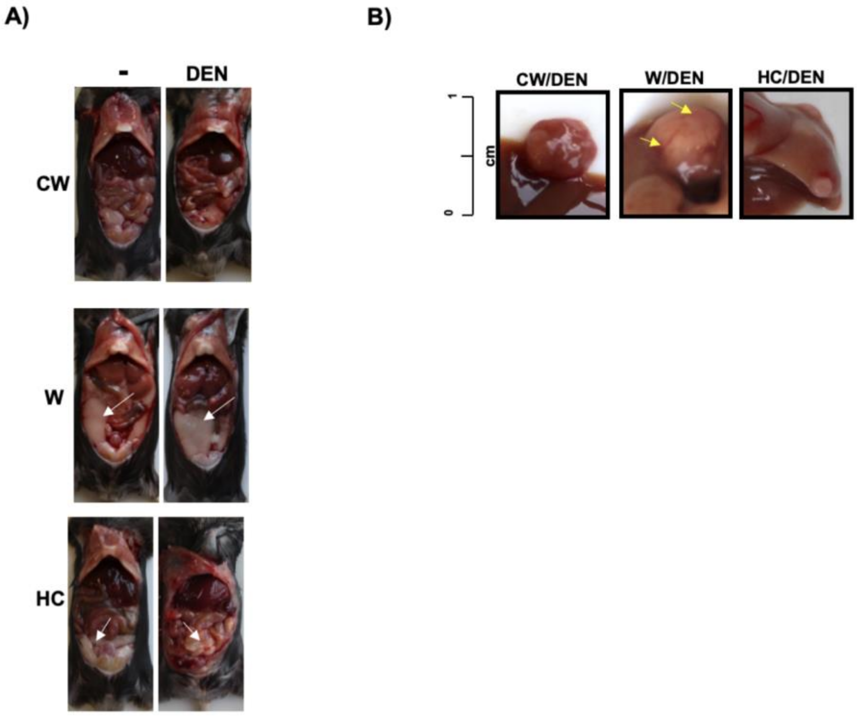

Figure S1. A) Gross inspection of mice fed with the different diets and treated or not with DEN. Representative image of at least eight animals, white arrows show adipose tissue. **B**) Tumor gross inspection of mice under different diets. Representative image of at least eight livers, yellow arrows show vascularity. Images are representative of at least eight mice.

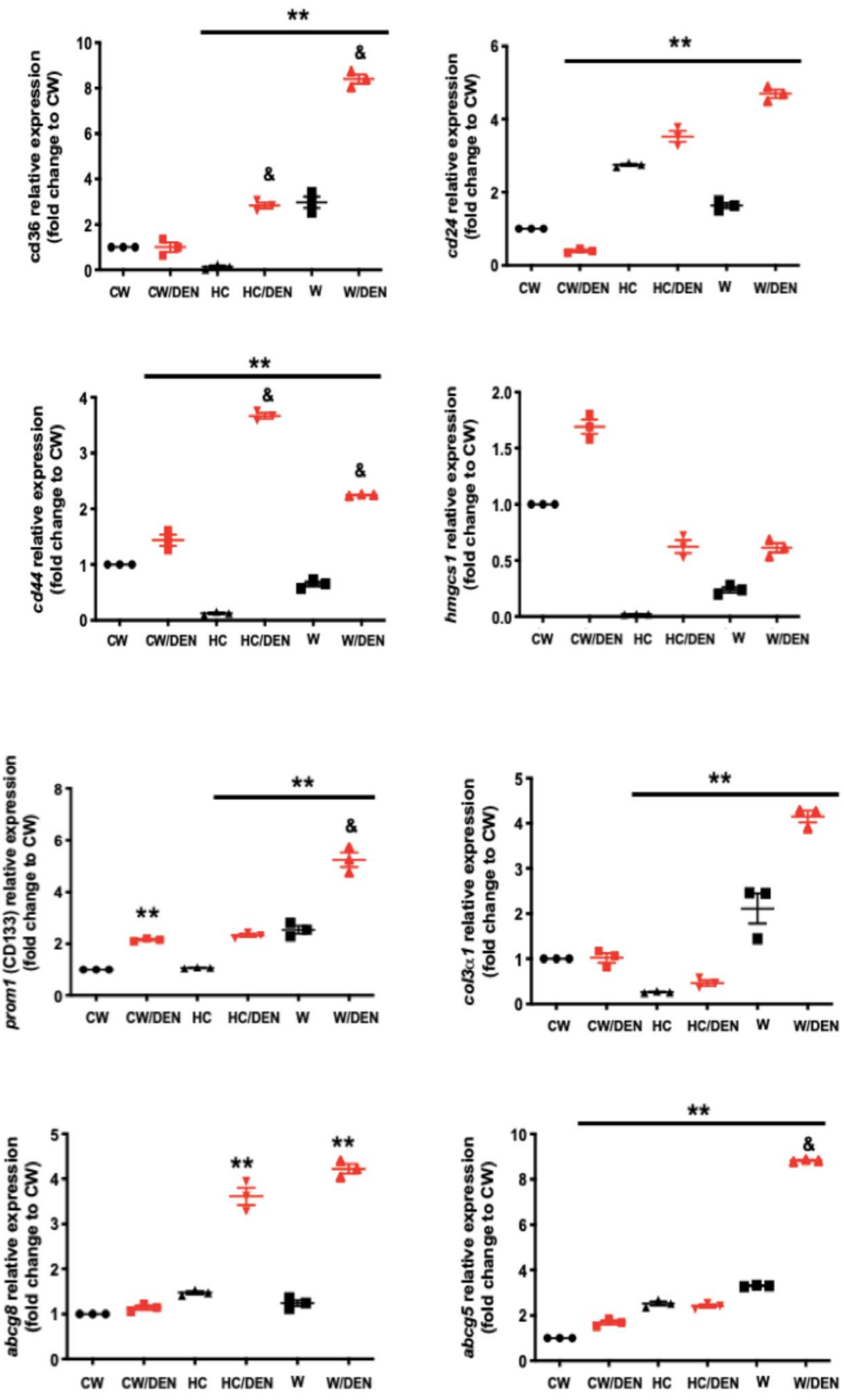

Figure S2. Expression of eight randomly selected genes in samples not included in the RNA-seq, as technical and biological validation. Each graph plots the individual data points, the superimpose horizontal line indicates the arithmetic mean and error bars showing ± SEM. ** $p < 0.01$ vs CW group; & $p < 0.05$ vs diet control.

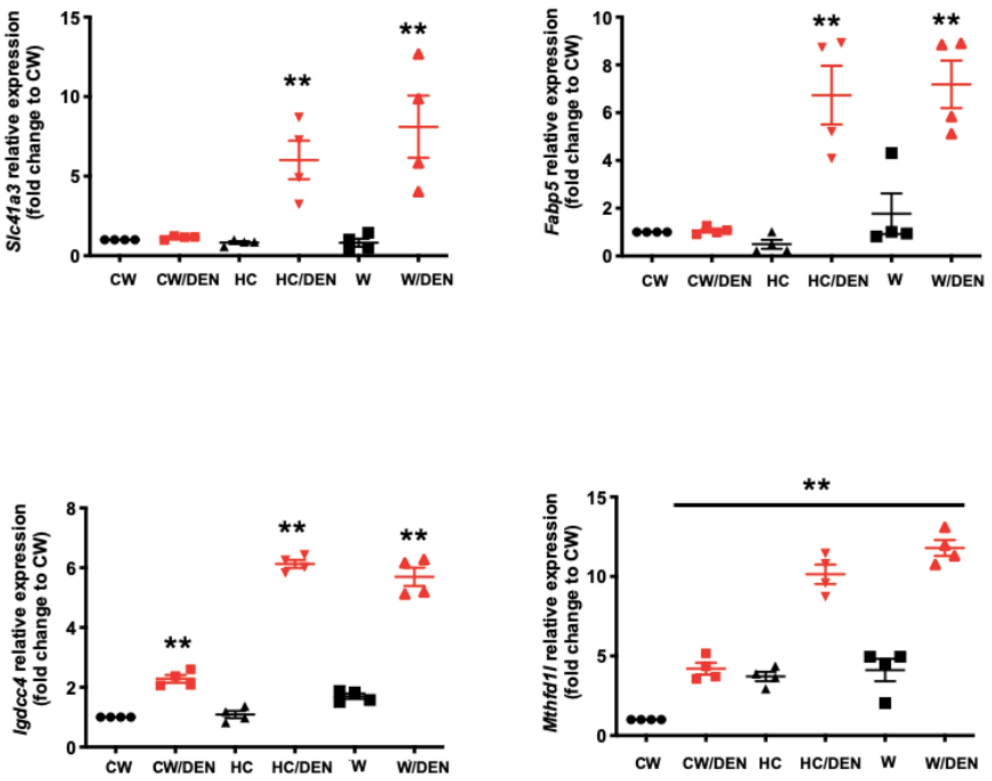

Figure S3. Expression of the 4 genes differentially overexpressed related to high lipid overload with potential relevance in tumor promotion: Mthfd1, Slc41a3, Fabp5, Igdcc4. Each graph plots the individual data points, the superimpose horizontal line indicates the arithmetic mean and error bars showing ± SEM. ** $p < 0.01$ vs CW group.

Table S1. Mice groups under different diets and treatments.

Group Number	Diet	DEN Single dose (10 ug/kg, ip)	Group ID	Number of Animals (*n*)
1	Regular Chow	-	CW	8
2	Regular Chow	+	CW/DEN	8
3	Western	-	W	8
4	Western	+	W/DEN	8
5	High cholesterol 1%	-	HC	8
6	High cholesterol 1%	+	HC/DEN	8

Table S2. Primers used in the study.

Gene Name	Ensembl ID	Primer Sequence
Cd24	ENSMUSG00000047139	5′-GCTCCTACCCACGCAGATTT-3′ (20)
		5′-TGGTGGTAGCGTTACTTGGA-3′ (20)
Prom1	ENSMUSG00000029086	5′-GAAAAGTTGCTCTGCGAACC-3′ (20)
		5′-TCTCAAGCTGAAAAGCAGCA-3′ (20)
Cd44	ENSMUSG00000005087	5′-TGGATCCGAATTAGCTGGAC-3′ (20)
		5′-AGCTTTTTCTTCTGCCCACA-3′ (20)

Col3α1	ENSMUSG00000026043	5′-GTGTCAAAGGTGAACGTGGC-3′ (20)<br>5′-ATCCCAGCAATCCCAAGTGG-3′ (20)
Slc41a3	ENSMUST00000032177	5′-AACAGCCAGACCTTTGTGGTGC-3′ (20)<br>5′-GATGCAGTGGTTGTCAGGATCC-3′ (20)
Fabp5	ENSMUST00000029046	5′-GGTGCATTGGTTCAGCATCAGG-3′ (20)<br>5′-TCATAGATCCGAGTACAGGTGAC-3′ (20)
Igdcc4	ENSMUST00000035499	5′-CGCACACCTGAATGTCAACTCC-3′ (20)<br>5′-CGTGGATGTCTCCAGTCAAGTG-3′ (20)
Mthfd1l	ENSMUST00000043735	5′-GCCATGGATGCCTTAAGTTC-3′ (20)<br>5′-AGCCAGGTAAACGAGTGAGC-3′ (20)
Cd36	ENSMUST00000082367	5′-CCTTAAAGGAATCCCCGTGT-3′ (20)<br>5′-TGCATTTGCCAATGTCTAGC-3′ (20)
Hmgcs1	ENSMUST00000179869	5′-TTTGATGCAGCTGTTTGAGG-3′ (20)<br>5′-CCACCTGTAGGTCTGGCATT-3′ (20)
Abcg8	ENSMUST00000045714	5′-CCTGATCCGTCGTCAGATTT-3′ (20)<br>5′-CCATGGCCGTAGTAAAGGAA-3′ (20)
Abcg5	ENSMUST00000066175	5′-CGTCCAGAACAACACGCTAA-3′ (20)<br>5′-GCAGCATCTGCCACTTATGA-3′ (20)
Rps18	ENST00000414228	5′-TGTGGTGTTGAGGAAAGCAG-3′ (20)<br>5′-TCCCATCCTTCACATCCTTC-3′ (20)

Table S3. Antibodies used in the study.

Antibody	Brand	Catalog number	Dilution
CD34	Abcam	ab81289	1:1000
F4/80	Abcam	ab60343	1:1000
CD206	Santa Cruz	sc-376232	1:250

Table S4. Gene expression profile of W/DEN vs CW/DEN.

Symbol	log2FoldChange	pvalue	padj
**Lcn2**	6.144249476	3.45E-37	7.73E-35
**Klrb1b**	6.02917133	1.11E-16	6.13E-15
**Cyp2a4**	6.005827794	3.82E-63	2.57E-60
**Apoa4**	5.998886702	2.12E-228	3.42E-224
**Gpc3**	5.982100042	5.65E-14	2.34E-12
**Gldn**	5.970045534	2.88E-16	1.51E-14
**Ly6d**	5.92348089	1.03E-13	4.09E-12
**5330417C22Rik**	5.584570455	6.93E-26	8.27E-24
**Akr1c18**	5.429597703	5.65E-14	2.34E-12
**Mmp12**	5.416961539	2.99E-14	1.28E-12
**Tspan8**	5.323519633	7.47E-20	5.57E-18
**Kif5c**	5.323052085	8.00E-11	2.28E-09
**Cacna1b**	4.696180458	4.37E-11	1.29E-09
**Gm14936**	4.627904898	3.66E-10	9.46E-09
**Cd63**	4.576152649	1.61E-15	7.70E-14
**Adcy1**	4.409834832	8.07E-23	7.60E-21

**Serpina12**	4.360637563	1.47E-93	3.96E-90
**Il1rn**	4.355639448	2.92E-10	7.70E-09
**Mep1b**	4.27501959	1.33E-07	2.21E-06
**Gpnmb**	4.237963381	1.38E-13	5.39E-12
**Cdh17**	4.185554666	4.76E-09	1.01E-07
**Ifi27l2b**	4.159263621	3.34E-22	3.01E-20
**Rgs16**	4.156732381	1.62E-26	2.06E-24
**Aebp1**	4.152364362	2.22E-15	1.05E-13
**Fgf21**	4.144283377	2.44E-10	6.51E-09
**Cyp2b13**	4.056988206	1.05E-08	2.13E-07
**Psat1**	4.051031065	3.79E-07	5.79E-06
**Gria3**	4.015795605	9.03E-13	3.25E-11
**Pnpla5**	3.990753527	7.98E-09	1.65E-07
**Tlr1**	3.981567094	3.44E-13	1.30E-11
**Vmn2r3**	3.903778609	8.36E-08	1.46E-06
**Galnt6**	3.87819635	1.33E-12	4.71E-11
**Sgsm1**	3.874246865	4.89E-10	1.24E-08
**Mmp13**	3.872872042	1.56E-08	3.09E-07
**Nrg1**	3.860096689	3.95E-08	7.28E-07
**Nid1**	3.846041182	1.50E-164	8.04E-161
**Afp**	3.822330051	3.09E-24	3.28E-22
**Ocstamp**	3.800300586	1.89E-14	8.19E-13
**Shc2**	3.796698238	9.08E-09	1.85E-07
**Fam83a**	3.751915473	1.96E-07	3.17E-06
**Fst**	3.736884935	5.20E-11	1.52E-09
**Tmem54**	3.607996424	4.29E-07	6.47E-06
**Esm1**	3.603017942	3.58E-09	7.82E-08
**Fstl4**	3.523169199	3.33E-06	4.23E-05
**Spink1**	3.521534323	9.87E-07	1.40E-05
**Lpar2**	3.515135681	9.05E-08	1.56E-06
**Slc24a3**	3.466003116	5.85E-06	7.07E-05
**Crybg2**	3.459202163	2.28E-10	6.11E-09
**Itih5**	3.456801018	7.12E-37	1.57E-34
**Ccdc120**	3.436843765	2.05E-06	2.73E-05
**Itgax**	3.428892088	3.91E-10	1.01E-08
**Ptpre**	3.415252249	3.39E-15	1.58E-13

**B4galt6**	3.413139284	2.21E-17	1.32E-15
**Dab1**	3.411976156	1.84E-06	2.48E-05
**Dusp5**	3.403156029	4.26E-07	6.43E-06
**Lpl**	3.379294591	5.62E-39	1.37E-36
**Rad51b**	3.350889497	8.07E-08	1.41E-06
**Stap1**	3.333851029	1.47E-07	2.44E-06
**Clec7a**	3.322445914	1.36E-14	5.94E-13
**Mtmr11**	3.320308971	3.46E-12	1.18E-10
**Kazald1**	3.319635151	8.19E-06	9.57E-05
**Spp1**	3.30271085	1.85E-76	2.30E-73
**Golm1**	3.29253552	7.04E-18	4.47E-16
**Igfbp1**	3.281109685	7.00E-17	3.94E-15
**Cacna1c**	3.133815428	6.05E-09	1.26E-07
**Neurl1a**	3.115707478	1.97E-06	2.64E-05
**Abcd2**	3.113308463	4.28E-44	1.28E-41
**Tff3**	3.111638191	6.74E-07	9.80E-06
**Ntrk2**	3.096815611	1.22E-06	1.71E-05
**Gm13387**	3.093223938	1.17E-08	2.36E-07
**Trem2**	3.084582288	7.70E-07	1.11E-05
**Mpped1**	3.084508324	1.11E-05	0.00012532
**Tmem86a**	3.083149209	3.94E-19	2.81E-17
**Rasal1**	3.078432784	4.43E-05	0.00043346
**Tlr13**	3.037861586	4.65E-07	6.94E-06
**Ipcef1**	3.002340096	9.61E-08	1.65E-06
**Igsf23**	2.993160174	2.67E-05	0.00027774
**Phgdh**	2.967018996	3.65E-07	5.59E-06
**Zfp57**	2.95800455	7.73E-08	1.36E-06
**Saa1**	2.939575243	5.82E-15	2.67E-13
**Rims2**	2.934984852	3.41E-09	7.46E-08
**Tnfrsf12a**	2.929747583	1.38E-14	6.02E-13
**Cd14**	2.913639412	1.29E-07	2.17E-06
**Scd2**	2.898182419	1.54E-12	5.41E-11
**Mpeg1**	2.880338559	6.43E-50	2.53E-47
**F2rl1**	2.874831327	8.03E-05	0.00074066
**Aspm**	2.864264918	8.47E-08	1.47E-06
**Itga2**	2.856246294	1.50E-07	2.48E-06

**Ddr1**	2.851998527	7.26E-08	1.28E-06
**Osbpl3**	2.843868969	4.45E-47	1.49E-44
**Lyve1**	2.835994713	9.52E-18	5.99E-16
**Cntnap1**	2.795180228	9.03E-16	4.45E-14
**Ptges**	2.779583209	7.48E-05	0.0006955
**Col1a1**	2.757587669	5.89E-30	9.49E-28
**Cdhr2**	2.748217767	6.50E-05	0.00061257
**Ccl6**	2.744812808	1.28E-08	2.58E-07
**Serpine1**	2.741809992	8.27E-06	9.64E-05
**Mthfd1l**	2.740371517	3.95E-06	4.95E-05
**Cers6**	2.736767355	8.29E-47	2.73E-44
**H19**	2.736502713	0.00067516	0.00475947
**Plch1**	2.731543411	5.29E-05	0.00051154
**Cenpf**	2.726498734	1.56E-08	3.09E-07
**Fhl3**	2.725248034	0.00019505	0.00162339
**Ephb2**	2.719101785	3.47E-05	0.00034986
**Abhd2**	2.718082453	7.11E-217	5.73E-213
**Scn8a**	2.714650239	2.42E-11	7.46E-10
**Cxcl9**	2.710852908	6.14E-11	1.77E-09
**Col4a3**	2.707018975	2.54E-13	9.72E-12
**Stambpl1**	2.705549328	3.43E-06	4.35E-05
**Smpd3**	2.699862618	7.93E-10	1.94E-08
**Cyp2b9**	2.697233386	4.59E-07	6.86E-06
**Tnfsf13**	2.684083382	1.63E-05	0.00017735
**Inhbb**	2.680214602	2.54E-06	3.32E-05
**Tmem229a**	2.674392393	3.60E-09	7.85E-08
**Anxa2**	2.673682501	1.99E-25	2.31E-23
**Tnfrsf21**	2.669529818	5.29E-17	3.02E-15
**Myo5c**	2.667275002	4.16E-06	5.17E-05
**Slc41a3**	2.666553276	1.10E-07	1.86E-06
**Btg2**	2.666094379	1.08E-26	1.40E-24
**Saa2**	2.664266191	2.12E-10	5.74E-09
**Elovl7**	2.662217301	5.90E-05	0.00056212
**Gdpd1**	2.659216565	7.45E-05	0.00069365
**Thbs1**	2.649147404	8.31E-13	3.03E-11
**Saa3**	2.643228075	1.16E-12	4.13E-11

**Drc1**	2.614207271	0.00028636	0.00228438
**Ccr2**	2.597060547	2.62E-07	4.14E-06
**Adgrv1**	2.594527936	2.09E-12	7.31E-11
**S1pr3**	2.59377194	1.06E-09	2.53E-08
**Sybu**	2.589083503	3.43E-05	0.0003462
**Amot**	2.583695843	2.60E-18	1.75E-16
**Cdkn2b**	2.555065459	0.00064067	0.00453819
**Zfp37**	2.537395818	4.04E-05	0.00040107
**Grk3**	2.524256683	6.93E-15	3.13E-13
**Cidec**	2.514018281	1.58E-16	8.58E-15
**Csf2rb2**	2.499307372	7.50E-08	1.32E-06
**Rgs2**	2.492034968	1.39E-06	1.91E-05
**Lgals3**	2.491956101	6.04E-07	8.88E-06
**Dbp**	2.489564018	9.64E-16	4.73E-14
**Pnpla3**	2.477318288	4.72E-06	5.80E-05
**Klf10**	2.472125509	1.97E-24	2.16E-22
**Hid1**	2.471388538	3.95E-09	8.48E-08
**Prom1**	2.460917493	5.14E-09	1.08E-07
**Psrc1**	2.456288419	0.00047032	0.00350232
**Ccl2**	2.439390774	0.00028694	0.00228786
**Cxcl1**	2.438947197	1.83E-20	1.43E-18
**Nucb2**	2.438211173	1.25E-11	3.97E-10
**Clmp**	2.433136874	2.08E-05	0.00022152
**Itpripl2**	2.419625796	7.15E-16	3.58E-14
**P2rx7**	2.419230887	5.19E-10	1.31E-08
**Cgref1**	2.419118996	2.76E-06	3.56E-05
**Itga6**	2.416334638	3.09E-18	2.04E-16
**Haus8**	2.410787192	1.66E-09	3.80E-08
**Vcam1**	2.39863435	6.21E-25	7.00E-23
**Scube1**	2.391077176	8.31E-05	0.00076263
**Mfge8**	2.389849401	1.17E-21	1.01E-19
**Apoc2**	2.383076442	4.14E-55	2.09E-52
**Gm5148**	2.382471451	0.0001877	0.00156938
**Ppl**	2.375343956	1.31E-17	8.08E-16
**Aqp7**	2.373914919	0.00011082	0.0009882
**Cyp4f16**	2.370935681	4.13E-07	6.26E-06

**Tyrobp**	2.370759052	1.26E-07	2.12E-06
**Aldh1l2**	2.361810969	0.00016621	0.00141031
**H2-Eb1**	2.361531506	5.89E-18	3.75E-16
**Clip2**	2.359651495	2.42E-08	4.66E-07
**Ear2**	2.357276425	1.51E-05	0.00016563
**Trpm4**	2.355584839	5.90E-06	7.12E-05
**Aldh18a1**	2.349727646	2.01E-05	0.00021461
**Gpc6**	2.346035965	1.09E-14	4.82E-13
**Thsd4**	2.345489729	8.73E-11	2.46E-09
**Cyp39a1**	2.341430756	1.45E-20	1.14E-18
**Blnk**	2.334954752	4.19E-06	5.20E-05
**Tmc5**	2.332712092	2.29E-05	0.0002415
**Per3**	2.330803004	6.76E-24	6.99E-22
**Osbpl10**	2.325039526	0.00092513	0.00624838
**Cdkn1a**	2.324510383	5.52E-05	0.0005313
**Fndc3b**	2.32427769	4.32E-46	1.37E-43
**Nipal1**	2.323776019	0.00024901	0.0020204
**Col1a2**	2.309602544	1.46E-22	1.35E-20
**A2ml1**	2.307187715	1.64E-09	3.76E-08
**Ly9**	2.302123004	6.50E-05	0.00061257
**Plxna3**	2.291804826	2.31E-06	3.06E-05
**Ctss**	2.289402355	8.95E-16	4.43E-14
**Sytl5**	2.288926722	5.38E-05	0.00051852
**Ttc39a**	2.280256824	3.17E-07	4.89E-06
**Mmp27**	2.277287756	9.04E-05	0.00082371
**Synj2**	2.271732654	1.78E-23	1.77E-21
**4930533B01Rik**	2.266982713	0.00023034	0.00188403
**Trib3**	2.264031273	3.72E-08	6.88E-07
**Pold4**	2.263891255	6.43E-08	1.15E-06
**Zfp9**	2.261444305	4.54E-07	6.79E-06
**Dhrs9**	2.247962773	8.38E-06	9.72E-05
**Cyb561**	2.241014518	4.60E-08	8.34E-07
**Lratd2**	2.214377289	2.84E-08	5.40E-07
**Slc9a7**	2.214083291	6.54E-05	0.00061624
**Myof**	2.213632764	9.42E-13	3.38E-11
**Mki67**	2.210226447	1.55E-08	3.07E-07

**Fabp5**	2.195864893	6.44E-07	9.40E-06
**Aig1**	2.177834152	1.12E-17	6.90E-16
**Ptafr**	2.164696117	0.00015537	0.00132535
**Cd300lb**	2.161787797	0.00045363	0.00339839
**Cdh1**	2.158855866	3.03E-10	7.95E-09
**Tmem71**	2.158021359	6.29E-05	0.0005951
**Pck2**	2.154974385	4.65E-05	0.00045334
**Ms4a7**	2.148683775	0.00094351	0.00634597
**Pls1**	2.144821815	0.00119133	0.0077228
**Lgmn**	2.139916522	2.52E-28	3.68E-26
**Clca3a1**	2.129845364	5.68E-16	2.89E-14
**Rgs1**	2.127915165	4.30E-05	0.00042282
**Uap1l1**	2.126011657	1.22E-09	2.88E-08
**Hist1h2bq**	2.122830485	0.00106766	0.00705443
**Ifngr1**	2.122695129	8.25E-33	1.53E-30
**Plaur**	2.120818652	7.77E-05	0.00071885
**Tenm4**	2.120215357	0.00031226	0.00245694
**Cd44**	2.118857539	6.70E-14	2.73E-12
**Chka**	2.112119284	2.64E-64	1.94E-61
**Ccnd1**	2.105009198	5.18E-13	1.91E-11
**Nxn**	2.098769673	1.85E-05	0.00019942
**Adamts14**	2.096663088	6.38E-08	1.14E-06
**Plau**	2.096088307	1.50E-05	0.00016505
**Rhoc**	2.087570833	2.76E-06	3.56E-05
**Tmsb10**	2.084767353	0.00055356	0.00402002
**Pak1**	2.084046828	8.28E-06	9.65E-05
**Clcf1**	2.082831361	3.31E-05	0.00033561
**Alox5**	2.080139032	3.04E-05	0.00031091
**Tnfrsf11a**	2.079841506	0.00053894	0.00393339
**Airn**	2.07778919	1.41E-10	3.88E-09
**Tnc**	2.076463773	4.73E-09	1.00E-07
**S100a11**	2.074586646	0.00155018	0.00967253
**Itga4**	2.0697042	6.05E-14	2.49E-12
**Top2a**	2.06920185	1.34E-06	1.85E-05
**Plat**	2.067626229	0.00019791	0.00164471
**Mt1**	2.065345283	3.83E-06	4.82E-05

**Sorl1**	2.06425559	3.01E-09	6.66E-08
**Cidea**	2.062952498	1.12E-07	1.89E-06
**Cav2**	2.059943922	8.12E-06	9.50E-05
**Rab3d**	2.059359288	5.98E-10	1.49E-08
**Racgap1**	2.058647513	0.00126727	0.00814629
**Sorbs2os**	2.057231232	2.20E-08	4.26E-07
**Slfn2**	2.051856371	0.00028136	0.0022489
**Armcx4**	2.049880067	2.73E-07	4.29E-06
**Igdcc4**	2.04744231	4.06E-05	0.00040259
**Prrg4**	2.044328275	0.00022361	0.00183552
**Hmmr**	2.040418241	0.00077111	0.00534244
**Sh3pxd2b**	2.039313322	2.28E-08	4.42E-07
**Themis**	2.036090623	3.09E-07	4.80E-06
**Gprc5b**	2.035933862	2.39E-05	0.00025107
**Apcs**	2.034057776	2.26E-15	1.07E-13
**Armcx3**	2.033675608	1.48E-07	2.45E-06
**Mycn**	2.032896971	0.00014263	0.0012316
**Arhgef2**	2.032158894	1.48E-15	7.09E-14
**Ifi44**	2.028151729	2.98E-05	0.00030644
**Tbc1d31**	2.026138649	8.34E-21	6.72E-19
**Cybb**	2.021824646	5.91E-12	1.95E-10
**01-Mar**	2.017332258	2.90E-05	0.0002996
**Gm3362**	2.016537573	0.00041079	0.00311951
**Eda2r**	2.013191446	0.00030824	0.00243034
**Sectm1a**	2.011485935	0.00149449	0.00937516
**Alcam**	2.008441098	4.70E-49	1.76E-46
**Cd300a**	2.003783179	1.01E-06	1.43E-05
**Hist1h2ao**	2.002258246	0.00133926	0.0085211
**Zfp641**	1.999180812	0.00022993	0.00188171
**H2-Aa**	1.996746996	1.32E-19	9.74E-18
**Slc20a1**	1.993564182	1.29E-22	1.20E-20
**H2-Ab1**	1.990283577	4.48E-11	1.32E-09
**Unc5b**	1.981868871	7.13E-11	2.04E-09
**Itgb2**	1.981533095	3.09E-09	6.82E-08
**Tc2n**	1.9793893	2.93E-09	6.48E-08
**Zfp462**	1.976662646	5.57E-08	1.00E-06

**Fkbp11**	1.96774403	2.73E-08	5.20E-07
**Laptm5**	1.966820582	8.67E-11	2.45E-09
**Hspa1b**	1.9661202	2.18E-05	0.00023133
**Sirpa**	1.963354166	4.93E-13	1.83E-11
**Atf3**	1.95865219	0.00042894	0.00323451
**Adam8**	1.956669092	0.00035234	0.00273392
**Capg**	1.949540172	0.00049362	0.00365158
**Steap4**	1.94522797	9.49E-51	3.92E-48
**Cpe**	1.94223659	0.00050507	0.00372156
**Ms4a6c**	1.942105434	3.04E-05	0.00031135
**App**	1.94074764	2.54E-51	1.11E-48
**Tent5a**	1.940277244	2.69E-09	5.98E-08
**Myo1f**	1.939988834	2.05E-07	3.30E-06
**Bcl2l11**	1.937941196	9.21E-11	2.59E-09
**Slamf8**	1.935729312	0.00040128	0.00306029
**Slc16a6**	1.930288195	6.42E-11	1.84E-09
**Kdelr3**	1.927223109	0.00019632	0.00163319
**Cd74**	1.926352295	5.83E-17	3.31E-15
**Slc7a7**	1.925715603	1.08E-07	1.83E-06
**Gas2l3**	1.918747359	2.86E-06	3.67E-05
**Osmr**	1.918352355	8.07E-13	2.94E-11
**St6gal1**	1.917615058	4.04E-31	6.92E-29
**Hk2**	1.916973912	4.42E-05	0.00043328
**Trim68**	1.916315378	0.00056074	0.00406122
**Col3a1**	1.916063709	1.46E-18	1.01E-16
**Rnu1b6**	1.91557664	0.00020205	0.00167562
**Cxcl10**	1.91463833	0.0007257	0.00506704
**Cln6**	1.912779879	2.28E-05	0.00024038
**Lepr**	1.909726431	1.36E-08	2.73E-07
**Arhgap22**	1.906662644	0.00106335	0.00702881
**Vill**	1.903307713	0.00134348	0.00854252
**Pdlim7**	1.900953814	3.88E-06	4.87E-05
**Ncapg2**	1.900608338	5.76E-05	0.00055066
**Abcc12**	1.892579273	0.00109838	0.00721554
**Emp1**	1.89093594	0.00024446	0.00198847
**Glis3**	1.888757469	2.34E-06	3.09E-05

**C3ar1**	1.877795408	1.39E-05	0.00015347
**F630028O10Rik**	1.874170067	5.12E-06	6.25E-05
**Cenpe**	1.873568767	9.85E-05	0.00089195
**Gpc1**	1.86257432	8.71E-16	4.32E-14
**Slc35f2**	1.856740844	0.00087031	0.00592028
**Tceal8**	1.849975181	5.62E-05	0.00053919
**Rbp1**	1.846889149	2.08E-05	0.00022188
**Ccnb2**	1.846860474	0.00081271	0.00559698
**Iqgap1**	1.845246997	1.49E-18	1.02E-16
**Igfbp3**	1.839313448	3.48E-16	1.79E-14
**Myo7b**	1.839026312	0.00042104	0.00318238
**Slc7a1**	1.826578349	0.00030299	0.00239568
**Soat1**	1.825959002	8.31E-09	1.71E-07
**Cd24a**	1.823651336	0.00040953	0.00311289
**A930019D19Rik**	1.809683376	3.10E-05	0.00031624
**Ckap4**	1.807038091	9.97E-06	0.00011415
**Caprin2**	1.80527918	5.95E-05	0.00056637
**Tpm1**	1.80377147	6.91E-23	6.56E-21
**2010003K11Rik**	1.799639339	1.81E-06	2.44E-05
**Unc119**	1.792666338	1.84E-05	0.0001986
**Ubd**	1.791322124	0.00135414	0.00859166
**Spaca6**	1.791203593	1.27E-13	4.96E-12
**Bco1**	1.788219605	2.99E-06	3.83E-05
**Anln**	1.788039817	0.00100133	0.00669575
**Klf6**	1.78493858	5.72E-10	1.43E-08
**Parvg**	1.782726632	0.00074802	0.00520254
**Pcdh17**	1.779197215	3.24E-07	4.99E-06
**Clec4a2**	1.779103037	0.00083926	0.00575276
**Abcg1**	1.778288222	5.46E-10	1.37E-08
**Plagl1**	1.777932872	0.00160737	0.00995896
**Nrg4**	1.77414325	1.02E-07	1.74E-06
**Bcl6**	1.768638177	1.08E-14	4.81E-13
**Klhl13**	1.768280147	2.22E-05	0.00023531
**Ypel4**	1.763981168	0.00046319	0.00345879
**Sla**	1.761567168	9.15E-06	0.00010562
**Ptprc**	1.757973018	3.20E-14	1.37E-12

**Rcan3**	1.754065037	0.00085398	0.00583135
**Pla2g7**	1.752421985	9.48E-05	0.00086069
**Gspt2**	1.748023439	0.0008026	0.00553679
**Ms4a6b**	1.747797594	0.00034304	0.00267565
**Cstb**	1.74747649	1.86E-05	0.00020055
**Plekha1**	1.744993802	3.06E-16	1.59E-14
**Pfkfb4**	1.743930067	0.00044105	0.00331344
**Ikbip**	1.743825262	1.98E-05	0.00021153
**Kbtbd11**	1.743478334	0.00063301	0.00449858
**Axl**	1.740597955	7.51E-15	3.38E-13
**Piga**	1.737901899	2.45E-08	4.72E-07
**Cd68**	1.732690243	1.43E-06	1.97E-05
**Sh3bp4**	1.731174665	0.00018199	0.00153132
**Rtn4**	1.729241428	5.47E-22	4.80E-20
**Snn**	1.719382906	0.00107908	0.0071153
**Itgam**	1.718281949	0.00049928	0.00368564
**Tlr9**	1.717935175	0.00041333	0.00313735
**Psd4**	1.710828244	3.01E-06	3.85E-05
**Ptgfrn**	1.710004553	4.01E-06	5.00E-05
**Ica1**	1.709254199	3.50E-07	5.38E-06
**Cd300lf**	1.702908546	0.00010494	0.00094094
**Fam217b**	1.701728844	0.00103156	0.00686374
**Enc1**	1.699820951	2.14E-08	4.16E-07
**Zfp608**	1.699079126	3.70E-08	6.86E-07
**Btc**	1.695263294	0.00053319	0.00390018
**Ly6e**	1.694983905	4.78E-23	4.61E-21
**Pdgfb**	1.694637765	0.00098226	0.0065764
**Cd53**	1.689134827	4.88E-06	5.98E-05
**Kif20b**	1.684215837	0.00076769	0.0053233
**Hpgds**	1.68315584	0.0002706	0.00217259
**Prrg1**	1.681592546	0.00114224	0.00744651
**Fgl2**	1.681093424	1.77E-07	2.90E-06
**Fam83g**	1.681033243	0.00088427	0.0060026
**Fblim1**	1.680009327	0.00089344	0.00605214
**Gbp2**	1.677439338	0.00011519	0.00101985
**Prc1**	1.673819664	5.53E-05	0.00053172

**Lrrtm2**	1.662415807	4.33E-08	7.93E-07
**Srrm4**	1.66210021	8.84E-06	0.00010228
**Tlr2**	1.659099392	0.00034014	0.00265558
**Lyz2**	1.657093717	9.72E-08	1.67E-06
**Prr5l**	1.65565232	5.56E-05	0.0005341
**Tmem263**	1.654117806	8.88E-08	1.54E-06
**Igsf6**	1.651951577	6.44E-06	7.70E-05
**Cd93**	1.645221235	5.21E-13	1.92E-11
**Rps12**	1.644370157	3.67E-09	7.96E-08
**Ikbke**	1.643104711	5.64E-24	5.87E-22
**Pygb**	1.64155796	3.23E-08	6.06E-07
**Lama5**	1.64005384	8.92E-13	3.22E-11
**H2-Q1**	1.635572	9.92E-05	0.00089573
**Loxl4**	1.634431649	4.12E-08	7.57E-07
**Cyp4f18**	1.633269744	0.00142344	0.00898536
**Prss8**	1.632802232	1.06E-06	1.49E-05
**Coq8b**	1.632244801	2.05E-07	3.31E-06
**Cpne8**	1.631397431	7.69E-05	0.00071263
**Itpr3**	1.628442769	1.56E-06	2.13E-05
**Greb1l**	1.62573449	9.37E-08	1.61E-06
**Selenon**	1.62572821	0.000167	0.00141552
**Aph1b**	1.625297272	0.0010732	0.0070794
**Gm15408**	1.625278216	8.34E-05	0.00076529
**Plin4**	1.623215765	1.46E-05	0.00016046
**Bcl2l1**	1.623084685	2.23E-18	1.51E-16
**Morc4**	1.621735693	0.00074513	0.0051847
**1700017B05Rik**	1.621030608	4.17E-11	1.24E-09
**Fcgr4**	1.620143462	0.00025063	0.00203147
**Fcgr3**	1.619740264	0.000108	0.00096627
**Susd2**	1.616991857	0.00070616	0.00496065
**Cd5l**	1.615699815	1.06E-09	2.54E-08
**Uggt2**	1.614834387	2.99E-08	5.65E-07
**Abcb1a**	1.614001203	2.20E-07	3.53E-06
**Adgre1**	1.609933635	1.02E-07	1.75E-06
**Oas3**	1.606732667	0.00077887	0.00538922
**Mycl**	1.600726542	0.00042674	0.00322244

**Pgm1**	1.597383394	0.00023858	0.00194456
**Unc13b**	1.595531146	3.60E-15	1.67E-13
**Dcbld1**	1.594614225	2.69E-07	4.24E-06
**Csf2rb**	1.592620938	2.32E-06	3.07E-05
**Tspan2**	1.592297654	0.00124283	0.00802119
**Slc22a27**	1.581920989	0.00103359	0.00687439
**Arsg**	1.581686779	5.42E-14	2.26E-12
**Btg3**	1.578143455	0.00093527	0.00630633
**Myadm**	1.56999005	1.15E-13	4.57E-12
**Mllt3**	1.568715873	4.21E-07	6.36E-06
**H2-DMa**	1.567999905	0.00108182	0.00713046
**Tmc3**	1.566937452	0.00038357	0.00294192
**Vnn3**	1.562043352	1.03E-41	2.92E-39
**Wfdc2**	1.555166069	0.00014617	0.00125749
**Dnajc10**	1.554028189	3.48E-24	3.67E-22
**Sorbs2**	1.553961382	1.78E-21	1.49E-19
**Fxyd5**	1.552343058	0.00129353	0.00827877
**Sema4d**	1.547895525	0.00126872	0.00815238
**Ppbp**	1.547694569	0.00142799	0.00900709
**D1Ertd622e**	1.546583156	1.14E-06	1.60E-05
**St8sia4**	1.542981305	0.00071459	0.00500893
**Rbl1**	1.542763668	0.00014957	0.00128469
**Limch1**	1.54119807	0.00056272	0.00407187
**Orm2**	1.540224748	0.00056879	0.00411398
**Sel1l3**	1.538904802	2.03E-16	1.09E-14
**Tubb6**	1.536683625	1.04E-05	0.00011789
**Insyn2b**	1.535370596	0.00111504	0.00729571
**Gm19705**	1.535295995	0.00125924	0.00810436
**Rsad2**	1.53413326	2.60E-06	3.40E-05
**Slc30a4**	1.532742517	2.35E-08	4.55E-07
**Gramd1b**	1.528773819	1.06E-06	1.49E-05
**Anxa9**	1.527497329	0.00044862	0.003364
**Myo5a**	1.523390756	2.44E-05	0.00025595
**Icam1**	1.520323402	7.19E-08	1.27E-06
**Creb3l2**	1.520161564	1.98E-24	2.16E-22
**Kcnj8**	1.517887363	0.00015092	0.00129493

**Rhbdf1**	1.515857082	8.68E-08	1.50E-06
**Clec12a**	1.513848857	0.0002673	0.00215361
**Tm4sf4**	1.506569188	1.43E-21	1.22E-19
**Gsdme**	1.505992855	2.51E-11	7.69E-10
**Bcl2**	1.50484005	0.0004755	0.00353273
**Zfp704**	1.503826306	4.87E-14	2.05E-12
**Exd1**	-1.502979625	0.00013875	0.00120196
**Smad9**	-1.507109526	1.01E-07	1.73E-06
**Sugct**	-1.508335982	3.50E-11	1.05E-09
**Ces1f**	-1.51419683	1.80E-11	5.63E-10
**Alas2**	-1.51908263	4.05E-18	2.64E-16
**Pigr**	-1.52199712	2.42E-38	5.57E-36
**Dcxr**	-1.522835274	2.76E-13	1.05E-11
**C4a**	-1.528144017	3.09E-07	4.80E-06
**Serpina6**	-1.531102381	2.14E-27	2.89E-25
**Lipc**	-1.531316296	2.48E-19	1.79E-17
**D630024D03Rik**	-1.53163833	0.00052024	0.00381762
**Tat**	-1.532800963	0.00012377	0.00108679
**Hhip**	-1.538316922	0.00086958	0.00591783
**Neat1**	-1.540711373	9.51E-10	2.29E-08
**Apoa2**	-1.542317157	2.68E-31	4.75E-29
**Gsta3**	-1.544078905	3.35E-68	3.00E-65
**Cyp4a12a**	-1.546181399	4.97E-08	8.94E-07
**Coq8a**	-1.547653574	8.17E-17	4.56E-15
**Gstt2**	-1.550347065	1.42E-15	6.83E-14
**D630039A03Rik**	-1.563232201	9.75E-08	1.67E-06
**Snord85**	-1.563574713	2.80E-05	0.00029038
**Ndrg2**	-1.564873935	2.50E-59	1.49E-56
**Nfil3**	-1.565177474	1.06E-11	3.41E-10
**Abcb11**	-1.565225463	5.98E-09	1.25E-07
**Ugt2a3**	-1.566147615	2.78E-22	2.54E-20
**Socs2**	-1.569083822	6.86E-05	0.00064365
**Ces1d**	-1.574355443	1.80E-16	9.70E-15
**Cyp4f15**	-1.576484984	4.36E-84	7.81E-81
**Cyp3a44**	-1.578510695	0.00022632	0.00185529
**Asl**	-1.582250785	3.38E-14	1.44E-12

**Cyp2e1**	-1.586193446	3.76E-22	3.35E-20
**Fam184a**	-1.587019022	0.00094129	0.00633367
**Rdh19**	-1.589845576	0.00153235	0.0095791
**Mapk15**	-1.591556589	2.60E-13	9.90E-12
**Otc**	-1.591768395	2.15E-20	1.67E-18
**Ttc36**	-1.599581916	1.42E-40	3.87E-38
**Ido2**	-1.600074202	1.63E-21	1.38E-19
**Cyp2d9**	-1.608897148	9.22E-19	6.46E-17
**Serpina3k**	-1.612160401	4.44E-29	6.69E-27
**Rgn**	-1.616715	1.22E-23	1.25E-21
**Col13a1**	-1.622182609	5.16E-06	6.28E-05
**Ugt1a9**	-1.622221743	1.49E-09	3.45E-08
**Hdhd3**	-1.626151987	8.25E-06	9.63E-05
**Cndp1**	-1.628610561	0.00111306	0.00728574
**Foxa2**	-1.63152565	4.49E-08	8.17E-07
**Pah**	-1.643928446	8.77E-26	1.03E-23
**Gm4952**	-1.646327196	2.27E-24	2.44E-22
**Ugt2b1**	-1.66028266	4.53E-10	1.15E-08
**Slc27a5**	-1.671838992	1.17E-67	9.91E-65
**Ggt6**	-1.680851172	2.44E-11	7.50E-10
**Arntl**	-1.682758604	2.28E-11	7.05E-10
**Ces1c**	-1.687729627	8.71E-57	4.84E-54
**Chic1**	-1.691340954	4.17E-05	0.00041132
**Nat8f5**	-1.69508371	2.04E-08	3.97E-07
**1700042O10Rik**	-1.698088672	0.00020712	0.00170975
**Npr2**	-1.699137343	2.78E-35	5.82E-33
**Pecr**	-1.700458548	1.43E-27	1.99E-25
**Slc22a28**	-1.705659424	1.12E-10	3.11E-09
**Ces2c**	-1.706929049	5.34E-07	7.91E-06
**Tmem86b**	-1.707782526	1.08E-16	5.96E-15
**Onecut1**	-1.714356216	6.69E-05	0.00062891
**Gstt3**	-1.716953214	5.24E-06	6.38E-05
**Pms1**	-1.718844919	7.91E-16	3.95E-14
**Adck5**	-1.744199977	5.00E-13	1.85E-11
**Cyp2u1**	-1.749578559	2.93E-09	6.48E-08
**Lrit1**	-1.752202049	1.12E-14	4.92E-13

**Mup14**	-1.75470347	3.21E-12	1.10E-10
**Aox3**	-1.757658852	2.53E-28	3.68E-26
**Lifr**	-1.758307748	4.75E-20	3.61E-18
**Msmo1**	-1.75938414	1.88E-16	1.01E-14
**Car3**	-1.759838234	1.54E-09	3.54E-08
**Fndc1**	-1.760698578	0.00156701	0.00974936
**Arhgef37**	-1.763068396	1.32E-06	1.83E-05
**Moxd1**	-1.765054477	2.10E-07	3.39E-06
**Agxt**	-1.765523732	6.79E-42	1.95E-39
**Ahcy**	-1.772680596	1.83E-49	7.01E-47
**Cyp2d11**	-1.773822367	1.82E-07	2.96E-06
**Mbl2**	-1.774638021	3.99E-53	1.89E-50
**Mreg**	-1.776505296	8.62E-14	3.48E-12
**Mir1948**	-1.781519598	1.11E-07	1.88E-06
**Car5a**	-1.791766511	2.13E-11	6.60E-10
**E2f8**	-1.798855634	3.92E-05	0.00039058
**Ncam2**	-1.799544796	3.57E-07	5.48E-06
**Gm4737**	-1.804080553	6.20E-23	5.91E-21
**Mup10**	-1.809250142	3.16E-08	5.94E-07
**Rfx4**	-1.810840469	8.17E-06	9.56E-05
**Slco1a1**	-1.819725786	3.25E-14	1.38E-12
**LOC100862446**	-1.835449664	1.85E-08	3.62E-07
**Nlrp5-ps**	-1.84156466	1.25E-07	2.09E-06
**Clec2d**	-1.846750365	2.01E-112	6.47E-109
**Ddc**	-1.855231663	3.66E-23	3.60E-21
**Apol7a**	-1.858381076	1.30E-35	2.75E-33
**Igfbp2**	-1.859075878	1.22E-56	6.56E-54
**Serpina9**	-1.862064605	0.00046666	0.00347826
**Mup17**	-1.88252774	4.05E-06	5.06E-05
**Tmem254c**	-1.883308331	0.00019475	0.00162179
**Pdlim3**	-1.884197857	0.00071458	0.00500893
**4933404O12Rik**	-1.892015916	9.21E-05	0.00083776
**Cish**	-1.893373631	6.60E-06	7.86E-05
**Abat**	-1.895948169	5.93E-73	5.97E-70
**Sntg2**	-1.907906926	3.89E-09	8.38E-08
**Aldh1l1**	-1.908090079	2.09E-80	3.38E-77

**Pon1**	-1.91215168	8.15E-31	1.34E-28
**Cadm4**	-1.91506585	6.27E-06	7.52E-05
**Myh11**	-1.915317708	2.11E-13	8.12E-12
**Colgalt2**	-1.916583885	1.03E-05	0.00011757
**Idi1**	-1.924369302	4.81E-35	9.95E-33
**Mup-ps16**	-1.927909228	6.17E-14	2.53E-12
**Mup21**	-1.929111863	1.25E-10	3.47E-09
**Ugt3a1**	-1.931688984	1.22E-15	5.93E-14
**Bhmt**	-1.933259348	1.46E-15	7.05E-14
**Hal**	-1.936231756	1.41E-17	8.64E-16
**Scnn1a**	-1.938360443	4.34E-15	2.01E-13
**Car14**	-1.94458693	1.46E-09	3.38E-08
**Fcna**	-1.947313976	1.85E-10	5.02E-09
**Sucnr1**	-1.948433609	1.21E-07	2.03E-06
**Mup2**	-1.952015051	9.40E-07	1.34E-05
**Enho**	-1.954756807	4.16E-05	0.00041083
**Cd163**	-1.960535562	4.65E-12	1.56E-10
**Cyp4f14**	-1.962532691	4.74E-51	2.01E-48
**Tlcd2**	-1.975081888	9.82E-15	4.38E-13
**Cyp27a1**	-1.977431609	2.29E-139	9.24E-136
**Prlr**	-1.981679913	1.69E-26	2.12E-24
**Rapgef4**	-1.986678339	3.40E-16	1.76E-14
**Ugt2b38**	-1.993492259	3.53E-09	7.71E-08
**Ttc39c**	-2.001429242	1.49E-34	3.05E-32
**Ces1b**	-2.002041376	2.46E-48	8.80E-46
**S1pr5**	-2.01101317	4.15E-07	6.28E-06
**Avpr1a**	-2.01497132	3.82E-12	1.29E-10
**Cyp4a32**	-2.017018761	1.03E-29	1.59E-27
**Efna3**	-2.018347431	2.86E-06	3.67E-05
**Sult2a8**	-2.020260142	8.29E-26	9.83E-24
**Cyp2f2**	-2.026309993	2.25E-30	3.66E-28
**Grm8**	-2.028301651	4.86E-08	8.80E-07
**C7**	-2.03199112	9.19E-08	1.58E-06
**Mup3**	-2.037221714	2.00E-15	9.51E-14
**Nudt7**	-2.039082426	3.06E-20	2.33E-18
**Cyp4a12b**	-2.052612065	1.06E-14	4.71E-13

**9030616G12Rik**	-2.05545123	3.55E-08	6.61E-07
**Dpy19l3**	-2.058135066	4.98E-22	4.39E-20
**Akr1d1**	-2.062593688	8.36E-24	8.59E-22
**Akr1c19**	-2.07458014	5.92E-33	1.11E-30
**Mup15**	-2.079098112	6.95E-09	1.44E-07
**Hpd**	-2.098851421	1.55E-34	3.12E-32
**Cyp2c69**	-2.106945127	4.58E-06	5.64E-05
**Cyp2d12**	-2.134247265	2.85E-06	3.67E-05
**Noct**	-2.146729759	1.17E-12	4.16E-11
**Lhpp**	-2.152403695	1.75E-21	1.48E-19
**Ces1g**	-2.154538841	5.03E-57	2.90E-54
**Gm4956**	-2.179663126	4.21E-10	1.08E-08
**Nat8**	-2.18027007	4.94E-15	2.27E-13
**Neb**	-2.186176544	4.87E-55	2.38E-52
**Cyp3a11**	-2.188226919	2.33E-21	1.94E-19
**Cyp3a41b**	-2.194942353	1.97E-06	2.64E-05
**Dct**	-2.246517437	6.54E-06	7.80E-05
**Mup4**	-2.270854112	1.17E-09	2.77E-08
**Ces1e**	-2.274250353	2.80E-48	9.82E-46
**Ankrd33b**	-2.292472713	1.48E-27	2.02E-25
**Adh6-ps1**	-2.29252163	5.42E-24	5.67E-22
**Pkhd1l1**	-2.292716253	5.89E-05	0.00056117
**Trhde**	-2.298973524	6.64E-08	1.18E-06
**Cyp26a1**	-2.301549881	9.55E-11	2.67E-09
**Mup5**	-2.304445213	1.05E-13	4.18E-12
**Gsta2**	-2.326608176	1.69E-13	6.56E-12
**Cyp2c23**	-2.351717544	8.71E-80	1.28E-76
**Mup6**	-2.358659167	1.15E-16	6.31E-15
**Nnmt**	-2.359630547	7.51E-21	6.08E-19
**Selenbp2**	-2.364164725	1.25E-08	2.52E-07
**Slco1a4**	-2.368765469	9.21E-30	1.44E-27
**Ass1**	-2.390416304	2.51E-20	1.93E-18
**Cth**	-2.397802726	2.96E-21	2.45E-19
**Serpine2**	-2.410776075	8.64E-16	4.30E-14
**Cyp7b1**	-2.453120558	2.81E-19	2.02E-17
**Cyp2a5**	-2.457921434	5.99E-22	5.22E-20

**Slc13a2**	-2.51691014	2.78E-07	4.36E-06
**Tm7sf2**	-2.524953308	3.86E-34	7.60E-32
**Lrtm1**	-2.52727082	1.33E-07	2.21E-06
**Urad**	-2.530069634	4.49E-17	2.61E-15
**Serpina1e**	-2.571186137	2.46E-37	5.59E-35
**Cyp1a2**	-2.649073132	7.65E-37	1.67E-34
**Ces2a**	-2.650526677	5.00E-48	1.72E-45
**Igfals**	-2.658191643	3.82E-65	3.08E-62
**Oat**	-2.688720856	6.78E-31	1.13E-28
**Snhg11**	-2.707780525	2.74E-42	8.02E-40
**Gm5424**	-2.718655333	8.87E-32	1.59E-29
**Cyp8b1**	-2.737245799	2.38E-44	7.24E-42
**Srgap3**	-2.747121562	2.08E-12	7.26E-11
**Hamp**	-2.755967956	2.66E-46	8.58E-44
**Cyp3a16**	-2.769927747	8.41E-11	2.38E-09
**Mup16**	-2.799100418	2.03E-13	7.84E-12
**Gm12718**	-2.80433765	3.09E-11	9.32E-10
**Sult5a1**	-2.80718959	2.95E-16	1.54E-14
**Mup8**	-2.819923679	4.56E-17	2.63E-15
**Mup13**	-2.835894621	3.00E-16	1.56E-14
**Cyp4a14**	-2.867274916	2.97E-18	1.97E-16
**Capn8**	-2.93093182	2.37E-15	1.12E-13
**Pitx3**	-2.980496456	3.96E-07	6.02E-06
**Gnmt**	-3.020435265	4.48E-88	1.03E-84
**Capn11**	-3.046970827	5.36E-08	9.63E-07
**Cyp2c50**	-3.125256032	1.72E-40	4.63E-38
**Hacl1**	-3.197063753	9.71E-78	1.30E-74
**Cyp2c29**	-3.306978059	1.14E-28	1.69E-26
**Slc22a7**	-3.326308254	2.25E-38	5.25E-36
**Mup1**	-3.365817468	4.06E-17	2.37E-15
**Keg1**	-3.366449451	2.88E-31	5.05E-29
**Cyp4a10**	-3.385984073	2.85E-45	8.84E-43
**Cyp2c37**	-3.486340194	2.41E-62	1.56E-59
**Ces3b**	-3.593742494	1.47E-61	9.13E-59
**Cyp2c55**	-3.612160533	5.78E-16	2.93E-14
**Mup12**	-3.728477488	1.45E-24	1.60E-22

**Cyp2c54**	-3.892888884	4.83E-76	5.56E-73
**Hsd3b5**	-4.018599523	1.76E-34	3.50E-32
**Susd4**	-4.117351431	4.58E-52	2.05E-49
**Clec2h**	-4.248217248	1.15E-26	1.48E-24
**Cyp2c53-ps**	-4.262878134	9.46E-14	3.80E-12
**Mup7**	-4.370398726	1.70E-52	7.84E-50
**Cyp2b10**	-4.399872642	3.19E-23	3.16E-21

Table S5. Gene expression profile of HC/DEN vs CW/DEN.

Symbol	log2FoldChange	*p*-value	padj
**Saa2**	7.432689453	1.23E-55	2.71E-52
**Saa1**	6.924540616	8.61E-69	1.14E-64
**Lcn2**	6.127052739	5.30E-37	4.07E-34
**Moxd1**	5.064040715	6.34E-19	1.48E-16
**Orm2**	4.534215431	3.84E-20	1.08E-17
**Mt1**	4.054434467	3.21E-13	4.17E-11
**Per3**	4.017268886	1.87E-63	1.24E-59
**Dbp**	3.677768446	8.21E-29	4.35E-26
**Mt2**	3.658480259	2.91E-09	1.77E-07
**Mfsd2a**	3.347056711	4.82E-24	1.96E-21
**Orm3**	3.157770021	6.24E-15	9.72E-13
**Tmc5**	3.05873482	4.01E-07	1.46E-05
**Per1**	3.050589709	2.82E-16	5.04E-14
**Grem2**	3.036205707	1.27E-16	2.33E-14
**Apoa4**	2.999698609	1.99E-57	6.60E-54
**Fgl1**	2.942136775	3.98E-63	1.75E-59
**Smpd3**	2.899977005	6.65E-11	5.83E-09
**Rgs16**	2.884678692	1.71E-14	2.55E-12
**Alpk1**	2.868289008	9.93E-13	1.10E-10
**Ciart**	2.706357128	2.46E-07	9.65E-06
**Acpp**	2.703478006	2.21E-10	1.66E-08
**Fabp5**	2.701179097	1.58E-09	1.01E-07
**Usp2**	2.675796349	3.81E-13	4.80E-11
**Lpin1**	2.583084934	1.42E-12	1.54E-10
**Dnah1**	2.582424433	5.44E-06	0.00015697
**Il1r1**	2.573838693	9.53E-19	2.14E-16
**Xirp1**	2.551795277	7.50E-08	3.36E-06
**Cxcl14**	2.538433724	6.95E-06	0.00019372
**Isyna1**	2.523856858	3.05E-06	9.22E-05
**Slc13a5**	2.514468866	6.93E-07	2.45E-05
**Slc37a1**	2.456222446	1.00E-08	5.57E-07

**E030018B13Rik**	2.438119503	2.99E-08	1.50E-06
**Cpne8**	2.436546718	1.64E-08	8.64E-07
**Mthfd1l**	2.416418819	2.17E-05	0.00052431
**Slc7a15**	2.413885237	1.22E-08	6.71E-07
**Pnpla3**	2.379129508	8.21E-06	0.00022598
**Mmd2**	2.378420685	3.36E-05	0.00075696
**Chil3**	2.300166521	0.00026881	0.0042215
**Scara5**	2.267437065	7.40E-11	6.40E-09
**H2-Bl**	2.260689448	0.00015828	0.00280519
**C4a**	2.251152704	5.95E-14	8.57E-12
**Adam11**	2.243305668	5.86E-22	1.94E-19
**Per2**	2.233722943	6.48E-22	2.09E-19
**Cyp21a1**	2.233716958	4.52E-05	0.00096805
**Nos1ap**	2.22802671	7.15E-13	8.38E-11
**Tff3**	2.22099297	4.90E-05	0.00103716
**Thrsp**	2.209798539	1.66E-13	2.28E-11
**Gm18852**	2.208302496	0.00026509	0.00417298
**Serpina3n**	2.204558419	3.35E-50	5.54E-47
**Hcn3**	2.179283775	2.45E-08	1.24E-06
**Slc13a1**	2.176429169	0.00014075	0.00257593
**Prg4**	2.17465808	1.70E-35	1.19E-32
**Nrep**	2.165396299	7.37E-07	2.59E-05
**Cyb561**	2.15994217	1.97E-07	7.92E-06
**Lrg1**	2.159547936	2.69E-24	1.15E-21
**Upp2**	2.148330216	1.63E-21	5.15E-19
**Rad51b**	2.103701721	4.36E-05	0.00093888
**Cd300lf**	2.101716008	1.58E-05	0.00039538
**Socs3**	2.087851828	8.95E-07	3.03E-05
**Mpeg1**	2.074997456	5.57E-26	2.63E-23
**Nrg4**	2.073840387	1.83E-09	1.16E-07
**Acot11**	2.03756466	6.42E-08	2.99E-06
**Tef**	2.026380647	2.39E-34	1.44E-31
**Rnu1b6**	1.96689313	0.00014892	0.00269341
**Gm4070**	1.961258784	0.00018179	0.00312966
**Ifi27l2b**	1.947390906	1.65E-06	5.25E-05
**Coq10b**	1.946337807	8.01E-14	1.14E-11
**Gpcpd1**	1.936156757	3.18E-13	4.17E-11
**Myc**	1.923953542	3.52E-05	0.00078607
**Lepr**	1.899112608	3.20E-08	1.59E-06
**Slc43a1**	1.885710287	3.64E-08	1.76E-06
**Igdcc4**	1.884086883	8.08E-05	0.00158429
**Snx10**	1.863524879	2.91E-13	3.86E-11

**Esm1**	1.861249885	5.84E-05	0.00121096
**Apcs**	1.845035836	8.39E-13	9.58E-11
**Cyp7a1**	1.838223788	3.88E-12	3.95E-10
**Orm1**	1.834182846	2.78E-19	6.94E-17
**Scd1**	1.833631896	4.84E-10	3.46E-08
**Nmrk1**	1.823478818	9.99E-12	9.59E-10
**Esr1**	1.819019003	5.21E-10	3.71E-08
**Alas1**	1.813102716	1.41E-10	1.13E-08
**Rorc**	1.801553627	9.20E-21	2.71E-18
**Clca3a1**	1.784972374	8.58E-11	7.28E-09
**Lonrf1**	1.784782104	3.10E-07	1.18E-05
**Syne4**	1.783743558	0.00061267	0.00823471
**Mycl**	1.77276599	0.00018092	0.0031188
**Ly75**	1.758366541	3.42E-08	1.67E-06
**Eif4e3**	1.75320225	0.00015148	0.00272627
**Ccdc57**	1.747162838	3.31E-07	1.24E-05
**Agap2**	1.741711619	0.00014238	0.00259286
**Ptch2**	1.722739106	0.00040934	0.00589053
**Irak3**	1.717778292	0.00036821	0.00536775
**Slc41a3**	1.69744247	0.00040573	0.00584492
**Cobl**	1.692342619	1.38E-16	2.50E-14
**Bhlhe40**	1.675968122	2.50E-08	1.26E-06
**Pfkfb3**	1.675493444	4.96E-07	1.79E-05
**Tmem268**	1.672850135	9.31E-08	4.10E-06
**Cd5l**	1.657377087	5.59E-10	3.96E-08
**Slc3a1**	1.654531285	8.23E-14	1.16E-11
**Tmprss2**	1.653879776	0.00016951	0.00296062
**Steap4**	1.648861879	3.39E-35	2.24E-32
**Bcl6**	1.628126488	7.08E-12	7.00E-10
**Nampt**	1.625705654	6.20E-12	6.21E-10
**Pklr**	1.621963368	5.32E-13	6.34E-11
**Scd2**	1.60660189	6.87E-05	0.00138771
**Neil2**	1.600949695	0.00025998	0.00410731
**Gadd45g**	1.568994757	8.19E-05	0.00159832
**Adrb3**	1.566731178	1.09E-05	0.0002872
**Plk3**	1.566141398	9.65E-07	3.25E-05
**Tnip2**	1.555819509	8.42E-06	0.00023135
**Slc20a1**	1.553333958	4.18E-13	5.22E-11
**Tgm1**	1.549152992	5.30E-06	0.00015408
**Ly6e**	1.547975745	7.26E-19	1.66E-16
**Arid5a**	1.544794405	0.00037221	0.00540914
**Tifa**	1.543012096	3.73E-06	0.00011116

**Zbp1**	1.536526327	0.00030334	0.00462661
**Slc13a3**	1.521135647	1.95E-09	1.22E-07
**Map3k14**	1.520830017	2.65E-05	0.00061682
**Wfdc2**	1.500821962	0.00039336	0.00568529
**Hpx**	1.500718692	6.95E-24	2.70E-21
**Fstl1**	-1.513227737	0.00024307	0.00389122
**Mapk15**	-1.525607498	1.53E-08	8.12E-07
**Creg1**	-1.532125346	6.65E-11	5.83E-09
**Ypel2**	-1.53419641	5.89E-10	4.15E-08
**Hamp**	-1.536004759	2.27E-15	3.71E-13
**Eps8l2**	-1.546130794	7.77E-17	1.47E-14
**Ces2a**	-1.546378092	2.95E-17	5.92E-15
**Rab36**	-1.548868826	2.03E-09	1.26E-07
**Sdr9c7**	-1.560546873	1.83E-10	1.42E-08
**Ccn1**	-1.574210546	5.48E-06	0.00015714
**Msmo1**	-1.574302097	7.37E-13	8.56E-11
**Pctp**	-1.574634152	1.58E-12	1.68E-10
**Cyp51**	-1.591094054	2.76E-10	2.04E-08
**Timp2**	-1.595984501	9.08E-06	0.00024791
**Sdf2l1**	-1.602328355	3.73E-09	2.22E-07
**Avpr1a**	-1.626141331	2.30E-07	9.12E-06
**Mme**	-1.628311606	5.47E-16	9.52E-14
**Cyp1a2**	-1.643370353	4.83E-15	7.61E-13
**Gstm6**	-1.644346074	1.43E-08	7.67E-07
**Agxt**	-1.652506625	2.52E-26	1.29E-23
**Ces1b**	-1.66152036	4.89E-24	1.96E-21
**Tubb4b**	-1.664759092	6.53E-13	7.72E-11
**Ehhadh**	-1.665416668	6.14E-20	1.63E-17
**Aox1**	-1.693466298	4.23E-39	4.31E-36
**Cyp3a59**	-1.712274537	6.19E-16	1.06E-13
**Mir122**	-1.71966781	2.44E-05	0.00057684
**Gstm4**	-1.721271019	1.84E-10	1.42E-08
**Clstn3**	-1.722738129	1.37E-08	7.41E-07
**Tubb2a**	-1.723137731	5.49E-07	1.96E-05
**Hmgcs1**	-1.732604849	9.43E-08	4.12E-06
**Cidea**	-1.740641074	3.30E-05	0.00074733
**Srgap3**	-1.742345511	3.44E-05	0.0007723
**Crot**	-1.757645891	6.02E-38	5.69E-35
**Ppp1r3c**	-1.769335415	7.01E-05	0.00141018
**Abcc3**	-1.777037655	3.25E-26	1.59E-23
**Mpzl1**	-1.784927411	0.00025959	0.00410603
**Nsdhl**	-1.823409421	3.40E-09	2.03E-07

**Gm4952**	-1.848292484	1.48E-19	3.84E-17
**Vegfd**	-1.860704645	0.00037842	0.00549333
**Ephx1**	-1.902052013	6.54E-18	1.35E-15
**Gm4956**	-1.908304111	5.47E-06	0.00015713
**Meg3**	-1.931060453	0.00019575	0.00329677
**Gdf15**	-1.945039162	2.73E-10	2.03E-08
**G6pc2**	-1.964568499	0.00072446	0.0093806
**Slc13a2**	-1.976074021	0.00056874	0.00776238
**Gclc**	-1.999998391	9.30E-38	8.21E-35
**Clpx**	-2.004524322	1.56E-44	1.88E-41
**Mreg**	-2.022064963	8.49E-13	9.61E-11
**Cyp3a41b**	-2.061650633	9.81E-05	0.00188682
**Car3**	-2.09534793	7.52E-13	8.66E-11
**Ces1g**	-2.118553897	1.46E-46	1.93E-43
**Pitx3**	-2.142440054	0.00078094	0.00999561
**Lrfn3**	-2.202775407	2.72E-07	1.05E-05
**Csad**	-2.323180715	5.31E-35	3.34E-32
**Pdk4**	-2.343147394	1.40E-08	7.57E-07
**Raet1a**	-2.345492704	1.22E-09	7.94E-08
**Cyp2b10**	-2.348134144	3.32E-07	1.24E-05
**Myh11**	-2.387773811	1.47E-11	1.38E-09
**Gstm1**	-2.407624804	1.85E-37	1.53E-34
**Gsta4**	-2.414340431	2.89E-12	3.01E-10
**Cyp2c55**	-2.426784697	1.40E-07	5.87E-06
**Clec2h**	-2.434207741	4.60E-13	5.69E-11
**Gstm3**	-2.445407564	2.48E-10	1.85E-08
**Spon2**	-2.46982861	2.22E-08	1.14E-06
**Cyp3a44**	-2.507616152	1.71E-06	5.42E-05
**Gsta2**	-2.541652769	2.06E-10	1.58E-08
**Hsd17b6**	-2.728590227	8.27E-25	3.65E-22
**Cyp3a16**	-2.822072976	9.71E-07	3.26E-05
**Acta2**	-2.828444234	1.02E-07	4.41E-06
**Cyp4a10**	-2.888166655	2.35E-32	1.30E-29
**Arntl**	-3.034545458	1.49E-15	2.46E-13
**Cyp3a11**	-3.104204805	3.36E-41	3.71E-38
**Idi1**	-3.634828259	1.31E-33	7.55E-31
**Cyp4a14**	-5.09373492	3.40E-51	6.44E-48

Table S6. Gene expression profile shared by W/DEN vs HC/DEN.

Symbol	log2FoldChange HC/DEN	log2FoldChange W/DEN
**Lcn2**	6.127052739	6.144249476
**Apoa4**	2.999698609	5.998886702

**Ly6e**	1.547975745	5.92348089
**Ifi27l2**	1.947390906	4.159263621
**Rgs16**	2.884678692	4.156732381
**Esm1**	1.861249885	3.603017942
**Rad51b**	2.103701721	3.350889497
**Tff3**	2.22099297	3.111638191
**Saa1**	6.924540616	2.939575243
**Scd**	1.60660189	2.898182419
**Mpeg1**	2.074997456	2.880338559
**Mthfd1l**	2.416418819	2.740371517
**Smpd3**	2.899977005	2.699862618
**Slc41a3**	1.69744247	2.666553276
**Saa1**	7.432689453	2.664266191
**Dbp**	3.677768446	2.489564018
**Pnpla3**	2.379129508	2.477318288
**Tmc5**	3.05873482	2.332712092
**Per3**	4.017268886	2.330803004
**Cyb561**	2.15994217	2.241014518
**Fabp5**	2.701179097	2.195864893
**Mt1e**	4.054434467	2.065345283
**Igdcc4**	1.884086883	2.04744231
**Apcs**	1.845035836	2.034057776
**Slc20a1**	1.553333958	1.993564182
**Steap4**	1.648861879	1.94522797
**Lepr**	1.899112608	1.909726431
**Nrg4**	2.073840387	1.77414325
**Bcl6**	1.628126488	1.768638177
**Cd300lf**	2.101716008	1.702908546
**Cpne8**	2.436546718	1.631397431
**Cd5l**	1.657377087	1.615699815
**Mycl**	1.77276599	1.600726542
**Wfdc2**	1.500821962	1.555166069
**Orm2**	4.534215431	1.540224748
**Cyp3a5**	-2.507616152	-1.578510695
**Mapk15**	-1.525607498	-1.591556589
**Arntl**	-3.034545458	-1.682758604
**Msmo1**	-1.574302097	-1.75938414
**Ca3**	-2.09534793	-1.759838234
**Moxd1**	5.064040715	-1.765054477
**Agxt**	-1.652506625	-1.765523732
**Mreg**	-2.022064963	-1.776505296
**Myh11**	-2.387773811	-1.915317708

**Idi1**	-3.634828259	-1.924369302
**Ces1**	-1.66152036	-2.002041376
**Avpr1a**	-1.626141331	-2.01497132
**Ces1**	-2.118553897	-2.154538841
**Cyp3a4**	-3.104204805	-2.188226919
**Cyp3a7**	-2.061650633	-2.194942353
**Gsta5**	-2.541652769	-2.326608176
**Slc13a2**	-1.976074021	-2.51691014
**Cyp1a2**	-1.643370353	-2.649073132
**Ces2**	-1.546378092	-2.650526677
**Srgap3**	-1.742345511	-2.747121562
**Hamp**	-1.536004759	-2.755967956
**Cyp3a7**	-2.822072976	-2.769927747
**Pitx3**	-2.142440054	-2.980496456
**Cyp4a11**	-2.888166655	-3.385984073
**Cyp2c18**	-2.426784697	-3.612160533
**Clec2d**	-2.434207741	-4.248217248
**Cyp2b6**	-2.348134144	-4.399872642
